# CRISPRmap: an automated classification of repeat conservation in prokaryotic adaptive immune systems

**DOI:** 10.1093/nar/gkt606

**Published:** 2013-07-17

**Authors:** Sita J. Lange, Omer S. Alkhnbashi, Dominic Rose, Sebastian Will, Rolf Backofen

**Affiliations:** ^1^Bioinformatics Group, Department of Computer Science, Albert-Ludwigs-University Freiburg, Georges-Köhler-Allee 106, 79110 Freiburg, Germany, ^2^ZBSA Centre for Biological Systems Analysis, Albert-Ludwigs-University Freiburg, Habsburgerstr. 49, 79104 Freiburg, Germany, ^3^BIOSS Centre for Biological Signalling Studies, Cluster of Excellence, Albert-Ludwigs-University Freiburg, Germany and ^4^Center for non-coding RNA in Technology and Health, University of Copenhagen, Gronnegardsvej 3, DK-1870 Frederiksberg C, Denmark

## Abstract

Central to Clustered Regularly Interspaced Short Palindromic Repeat (CRISPR)-Cas systems are repeated RNA sequences that serve as Cas-protein–binding templates. Classification is based on the architectural composition of associated Cas proteins, considering repeat evolution is essential to complete the picture. We compiled the largest data set of CRISPRs to date, performed comprehensive, independent clustering analyses and identified a novel set of 40 conserved sequence families and 33 potential structure motifs for Cas-endoribonucleases with some distinct conservation patterns. Evolutionary relationships are presented as a hierarchical map of sequence and structure similarities for both a quick and detailed insight into the diversity of CRISPR-Cas systems. In a comparison with Cas-subtypes, I-C, I-E, I-F and type II were strongly coupled and the remaining type I and type III subtypes were loosely coupled to repeat and Cas1 evolution, respectively. Subtypes with a strong link to CRISPR evolution were almost exclusive to bacteria; nevertheless, we identified rare examples of potential horizontal transfer of I-C and I-E systems into archaeal organisms. Our easy-to-use web server provides an automated assignment of newly sequenced CRISPRs to our classification system and enables more informed choices on future hypotheses in CRISPR-Cas research: http://rna.informatik.uni-freiburg.de/CRISPRmap.

## INTRODUCTION

Acquired immunity in prokaryotes is directed by Clustered Regularly Interspaced Short Palindromic Repeats (CRISPRs) and their associated (Cas) proteins. This CRISPR-Cas system, present in many bacteria and most archaea, recognises and subsequently degrades exogenous genetic elements [for recent reviews see (1–3)]. The adaptive immune response is divided into three main phases: (i) ‘Adaptation’, the selection of short target segments (protospacers) from foreign DNA and the incorporation of their reverse complement sequence (spacers) into the organism’s active CRISPR locus between directly repeated sequences (repeats); (ii) ‘crRNA maturation’, expression of the CRISPR RNA (a leader followed by an array of repeat-spacer units) and subsequent processing of the transcript into mature RNA species, called crRNA; and (iii) ‘target interference’, invader DNA (4) or RNA (5,6) degradation at the respective protospacer, guided by the crRNA and a highly specific complex of Cas proteins such as Cmr (5) or Cascade (7).

CRISPR arrays are associated with diverse sets of Cas proteins. Therefore, several global classification systems of Cas subtypes have been introduced (8–10). In the literature, CRISPR-Cas systems are frequently characterised solely by the associated Cas-protein subtypes and relationships between repeats are rarely considered. Although this division into Cas-subtypes is generally effective, an accurate Cas-protein-based classification is complicated: First, CRISPR loci may include novel, chimeric, mixed subtypes or *cas* genes that are missing entirely (10–14). Second, it is not always obvious which *cas* genes are specific to a repeat-spacer array or Cas proteins could be shared between arrays (13). Finally, many of the cas genes belong to extremely diverse families (8,10).

We provide a comprehensive classification of all publicly available CRISPRs that is based solely on the sequence and structure evolution of repeats. The repeat-spacer array is the only element to be present in all systems and CRISPR-Cas systems are identified first by the existence of such an array. In contrast to the annotation of *cas* genes, repeat-spacer arrays are easily identified by programs such as CRISPRFinder (15) or CRT (16). The repeat is the central regulatory element in the CRISPR-Cas system, as it serves as a binding template for Cas proteins in all three phases of immunity. For these reasons, a systematic repeat-based classification is fundamental to further understand the function, diversity and phylogeny of CRISPR-Cas immune systems.

All clustering approaches are based on pairwise similarities: similarities between repeats are assumed to reflect conserved binding motifs and mechanisms. The binding affinity of Cas proteins is not only affected by the repeat sequence: a small hairpin structure is a key binding motif for Cas endoribonucleases in several systems (17–25). To correctly identify these structure motifs, our clustering is the first that is based not only on sequence—but also on structure—similarities. This approach is well-established for the identification and characterisation of structured non-coding RNA (ncRNA) (26–29). For these ncRNAs, the conservation of structure is often more important than sequence for the biological function (30,31). Although CRISPRs are partially structured ncRNAs, no structure-based clustering exists. To our knowledge, the only CRISPR-specific classification was performed on 349 bacterial and archaeal repeats in 2007 (32). Although structure motifs were identified, the underlying clustering was based purely on sequence and not structure similarity. An analysis of the archaeal domain, also based on only sequence similarities, was done more recently (12).

To provide a complete overview of the conservation of both unstructured and structured CRISPRs, we performed an independent sequence-based clustering to identify conserved sequence families. In addition, we combined identified structure motifs and sequence families with a hierarchical representation of sequence and structure similarities to generate a map that directly reflects relationships between classes and individual CRISPRs. This hierarchical CRISPRmap tree enables a fast comparison between CRISPRs of interest and previously published systems. Automated access to our data via an easy-to-use web server allows users to identify relative positions of both published and unpublished sequences. CRISPRmap is a valuable resource to elucidate and generalise functional mechanisms of CRISPR-Cas immunity.

We rigorously analysed clustering results and observed the following: First, identified structure motifs and automated Cas subtype annotations are consistent with experimentally verified work (18–23). Second, cleavage sites in relation to the structure motifs could be inferred from common features observed in the many articles on crRNA maturation (13,17–21,23–25,33–36). Third, sequence families exhibit varying patterns of repeat sequence conservation. Fourth, some type I and both type III Cas subtypes do not correlate with repeat and Cas1 evolution. Finally, examples of horizontal transfer events of CRISPR-Cas systems between bacteria and archaea are identified, supported by CRISPR conservation and Cas homology.

## MATERIALS AND METHODS

### CRISPR data

#### Repeats from all publicly available genome sequences

All currently available genome sequences were downloaded from the NCBI server (http://www.ncbi.nlm.nih.gov/) and the CRISPR databases, CRISPI (37) and CRISPRdb (38) (August 2012). Redundant genomes were removed. We predicted CRISPRs using the two most common programs, CRISPRFinder (15) and CRT (16). For both tools, we used parameters that corresponded to at least three repeats within an array; repeat and spacer lengths were set to 18–58 nt. Although repeats within one array are largely identical, they can contain some mutations, especially toward the 3′-end of the array. Thus, we used a single representative repeat of a CRISPR array by calculating the consensus sequence of all repeat occurrences. Finally, we merged the results from both programs and the CRISPR databases to form a non-redundant set of >3500 consensus repeats, which we refer to as REPEATS. Table 1 gives a summary of our REPEATS data set. The results from CRISPRFinder and CRT give no information on the correct strand orientation. Therefore, we predict the repeat orientation within our clustering approach.

**Table 1.** Summary of our REPEATS data set including all publicly available CRISPR arrays

Data descriptor	Archaea	Bacteria
Genomes	279	2289
Genomes with CRISPRs (%)	177 (63)	877 (38)
Plasmids	41	1286
Plasmids with CRISPRs (%)	14 (34)	76 (6)
CRISPRs	643	2884
Repeats per array (median)	3–190 (15)	3–1371 (12)
Repeat lengths (median)	20–44 (29)	19–48 (30)
Spacer lengths (median)	20–50 (38)	19–70 (35)

#### Set of repeats from Kunin *et al.* 2007 (32)

We downloaded the data set from the supplementary material of (32) and refer to it as REPEATS*_Kunin_*. This data set contains 271 bacterial and 78 archaeal sequences (349 in total). The orientations were predicted by the authors using previously published sequence features.

#### Set of archaeal repeats from Shah and Garrett 2011 (11)

We received 378 archaeal repeat sequences from Shah and Garrett that were the basis for the results in (11). The repeat orientations were manually verified by Shah and Garrett. We refer to this data set as REPEATS*_Shah_*.

### Identifying conserved structure motifs

Our procedure for identifying conserved, local, hairpin-structure motifs (referred to as structure motifs) in all CRISPRs involves a complex multi-faceted workflow.

#### Step 1—Pool of unstructured repeats

The procedure starts with a pool, *P_u_*, of repeats that have not been assigned to a structure motif. Initially *P_u_* contains our entire REPEATS data set. The orientation of each repeat is predicted by a graph-kernel-based machine-learning model (39), slightly modified to work on directed graphs. We trained the model on the REPEATS*_Shah_* data (using the 253 repeats that had <95% similarity to ones in REPEATS*_Kunin_*). Each repeat sequence is given as a directed graph, i.e., the nucleotides are represented by nodes, which are linked by directed edges indicating the particular orientation. To test the performance of our model, we applied it to the REPEATS*_Kunin_* data. Overall, we achieved a performance of 0.68 for the area under the receiver operating curve (ROC) with feature parameters radius *r* = 1 and distance *D* = 2. Because we did not achieve a perfect orientation prediction (mostly due to insufficient training data), we addressed this issue throughout our clustering process. Nonetheless, the model ensures that at least the majority of sequences are in the correct orientation for the first clustering steps.

#### Step 2—Generating a hierarchical cluster tree reflecting sequence and structure similarity

A hierarchical cluster tree *T_i_* for the current iteration *i* is generated from all sequences in *P_u_* using RNAclust (27). RNAclust uses a hierarchical clustering algorithm [UPGMA (40)] based on similarities calculated with a sequence-and-structure alignment program, LocARNA (27,29). Thus, the relationships in the resulting binary tree not only reflect sequence, but also structure similarity. For each node of the cluster tree, there exists a sequence-structure alignment with the respective predicted consensus structure as given by LocARNA.

#### Step 3—Selecting subtrees with CRISPR-like consensus structures

Starting from the root node in *T_i_*, each child node is traversed in hierarchical order until a CRISPR-like hairpin consensus structure is found at a certain node *t*. The consensus structure is *local* in the sense that it does not cover the entire repeat sequences. All repeats descending from node *t* are considered to form a candidate structure motif, 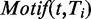, if the following requirements, derived from published repeat structures (17–23), are met: First, the consensus structure of 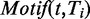 is a hairpin with a stack of at least 4 bp and no bulges or internal loops. Second, at least 10 repeat sequences fit to the consensus structure of the motif candidate. All repeats that do not fit to the consensus structure are removed from 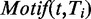. Third, the two child nodes of *t* must have compatible consensus structures. This means at least 75% of the base pairs must overlap with the consensus structure at *t*. The remaining child nodes of *t* are assigned to 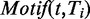 and the procedure is repeated until all nodes in *T_i_* have either been checked for—or have been assigned to—a structure motif.

#### Step 4—Supertree of only structured repeats

All repeats that have not been assigned to a structure motif are removed from the tree and are put back into the pool of unassigned repeats *P_u_*. All other repeats, which form one of the consensus structures, are put into a set *P_s_*. From this set *P_s_,* a *supertree*, *ST*(*i*), is generated by repeating Steps 2 and 3. Again repeats that do not conform to the criteria are removed and put back into the unassigned pool *P_u_*. This reclustering ensures the robustness of identified motifs.

#### Step 5—Merging supertrees

In one RNAclust run, we identify conserved structures of repeat sequences that are neighbouring in the cluster tree *T_i_*. To locate more distantly related repeat sequences that can still form a common consensus structure, we repeat the clustering with the remaining sequences in the pool *P_u_*. Consequently, Steps 2–4 are repeated for three iterations, resulting in three separate supertrees (*ST*_1_, *ST*_2_ and *ST*_3_) that are merged into one supertree, 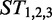. Merging starts with *ST*_1_. Because it is the result of the first iteration, it includes the largest and most well-conserved structure motifs. Each structure motif of the supertrees *ST*_2_ and *ST*_3_ is merged with *ST*_1_, one at a time. Due to the orientation uncertainty, we also attempt to merge the reverse complement sequences of the whole structure motif. Merging occurs by repeating Steps 2–4 and we use the orientation that results in the fewest number of repeat sequences being lost to *P_u_* in the merging process.

#### Step 6—Final cluster tree with structure motifs

We perform a last post-processing step to produce the final cluster tree with the structure motifs. For each structure motif, we calculate the consensus structure of the reverse complement repeat sequences. *GU* base pairs cannot form in the reverse complement orientation; therefore, we consider the orientation with the most stable consensus structure to be correct. We also check whether the reverse complement of a motif can be merged with another existing motif. Two biological features were used to check the orientations of entire motifs: the conserved 3′-end of repeats, *AUUGAAA(C/G)* and a majority of *A* instead of *U* nucleotides for archaeal sequences—as observed in the manually verified orientations in REPEATS*_Shah_*. If any changes were made in the orientation, Steps 2–4 are repeated. Note that changes to the input set can lead to changes in the resulting tree; therefore, our repeated runs of RNAclust ensure that most of the noise is removed and we only include stable structure motifs in our final result.

#### Improving the orientation of repeats

The identification of conserved structure motifs gives some evidence on the likely orientation of the repeats involved. For the unassigned repeats, however, we had no information to deduce the correct orientation. Therefore, we merged all structured repeats with the REPEATS*_Shah_* data and retrained our prediction model; we excluded repeats 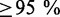 similarity with the test data. Again, we tested our model on the REPEATS*_Kunin_* data and achieved a substantial improvement with an area under the ROC of 0.82 in comparison with 0.68 previously. We subsequently used our retrained model to predict the correct orientation of the repeats remaining in the unassigned pool *P_u_*. Even if some orientations are still incorrect, this step ensures that the repeat orientations in our REPEATS data are consistent. To add the sequences that were previously in the incorrect orientation, we repeated Steps 1–6 with the improved orientation predictions.

### Clustering of repeat sequences into conserved sequence families

Repeat sequences were clustered into related families based on global sequence similarity using Markov clustering (MCL) (41,42). The MCL method is a popular method for clustering biological sequence data and was applied previously to CRISPRs (11,32). First, we calculated pairwise similarities with the Needleman–Wunsch alignment algorithm (43). These similarities (i.e., percent identities) were plotted (Supplementary Figure S1) and a reasonable cutoff of 65% identity was chosen to represent a significant similarity. Similarities below this value were explicitly set to zero to reduce noise. We ran the MCL program (downloaded from http://micans.org/mcl/) with an inflation parameter 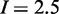. This parameter gave a good balance between the number of sequences assigned to a family and the conservation within a family. Only clusters with at least 10 repeat sequences were considered as a *conserved* sequence family.

We supplemented the Markov clustering with sequence profiles generated by CLUSTAL W (v. 1.83) (44). We used these profiles to reassign repeats to families to which they are sufficiently similar, as follows: Let 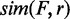 be the profile score of a repeat *r* compared with the profile of the family *F*, where 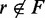. For each family, the minimum *F_min_* and maximum *F_max_* profile similarity was determined by removing each sequence from the family, recalculating the profile for the remaining sequences and determining the similarity score of the respective repeat to the profile. A repeat *r* was then assigned to a sequence family *F* if 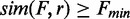 and the distance between 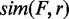 and *F_max_* is the minimum for all families. In total, 73 sequences were reassigned by the sequence profiles. The sequence conservation did not change significantly, but we were able to identify those few repeats that where missed by the MCL algorithm.

For each family, we generated sequence logos (Supplementary Figure S10 and Supplementary Tables S2–S19) by creating a multiple sequence alignment with the MAFFT program (45), version 6.4. The multiple sequence alignment was converted into a logo by WebLogo version 3 (46).

### Cas gene and Cas-subtype annotations

#### Annotations of all cas genes

Subtype-independent annotation of *cas* genes was performed on the entire chromosome or plasmid that harbours the respective CRISPR array. We applied the TIGRFAM models from Haft *et al.* (8,47) in combination with HMMER (48), but used the more recent *cas* gene names from Makarova *et al.* (10). A *cas* gene was annotated when one of its respective models was found with an E-value 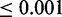. On our web server Web site, we offer a full table of *cas* gene annotations for each repeat, giving the minimum distance of that gene to the CRISPR array. For each sequence family and structure motif, we identified single *cas* genes that were associated with the majority of CRISPRs in the respective class (categories 50–69%, 70–89% and 90–100%); all *cas* genes on the entire chromosome or plasmid with the CRISPR were considered. Results are given in summary in Supplementary Tables S2–S19 and in full on the web server.

#### Cas subtype annotation from Makarova et al. 2011 (10)

The automatic annotation of subtypes is tricky owing to the fact that genes of multiple subtypes can be present in the genome, subtypes are often incomplete and it is not known if the *cas* genes must be within a certain distance of the CRISPR array. However, in many published CRISPR-Cas systems, the *cas* genes are located either directly upstream or downstream of the array (10). We used the following procedure that enabled a suitable trade-off between strictness and completeness of the annotations. We first compiled a list of signature *cas* genes that were unique to each type and subtype from (10). For each repeat, i.e. CRISPR array locus, we identified the closest subtype signature and then noted the distance of the respective type signature, if available. We plotted the distance of subtype and type signatures and determined a clear peak (at 14.5 kb) in their distances to their respective CRISPR array (Supplementary Figure S3). We considered a cutoff of 180 kb to represent a suitable distance from the CRISPR array; this cutoff corresponds to the 70th percentile of distances of the subtype signatures. A repeat is assigned to a subtype if both subtype and type signatures are within this distance. Note that with this approach, not all *cas* genes have to be present (or annotated).

#### Clustering of Cas1 proteins

Cas1 protein sequences were assigned to the closest CRISPRs if they were within 180 kb of the array (see Supplementary Figure S3 for cutoff explanation). These Cas1 proteins were again clustered using MCL (41,42) with default parameters. Here, pairwise sequence similarities were calculated with the local Smith–Waterman alignment algorithm (49) and percent identities <40% were set to zero to reduce noise. Only clusters with at least 10 proteins were considered.

### The CRISPRmap cluster tree

The tree was generated by RNAclust (27) and visualised with iTOL (50). In the tree, we see relationships based on sequence *and* structure similarity; however, when the repeat is unstructured, only the sequence similarity is considered. The encircling rings correspond to the following annotations (displayed as selected by the user): structure motifs, sequence families, Cas subtypes, phyla (taxonomy) and superclasses. The branches are coloured according to whether the CRISPRs were from bacteria or archaea.

### Web server input: adding new sequences

The user of our CRISPRmap web server can enter up to 300 CRISPR sequences in FASTA format and indicate whether the correct orientation is unknown and requires prediction. We use a multi-step procedure that has been optimised for speed to assign the given repeats to our structure motifs and sequence families. Further details are given in the Supplementary Methods S1.2.

## RESULTS AND DISCUSSION

### All available CRISPR sequences from bacteria and archaea

We obtained >3500 consensus repeat sequences from predicted CRISPR arrays in ∼2500 available genomes. This data set, referred to as REPEATS (see Table 1), is the most complete set of CRISPRs to date. We compared the REPEATS data set to previous work in Supplementary Figure S5.

### Structure motifs and sequence families

We performed a comprehensive search for both conserved sequence families and small CRISPR-like hairpin motifs, using *independent* approaches to allow for both structured and unstructured repeats. First, we partitioned CRISPRs into sequence families using Markov clustering, as in previous studies (11,32); in addition, we applied sequence profiles to refine the Markov clusters. We identified 40 conserved families. The mean pairwise sequence identity of 68–96% (avg. 82%) reflects a high level of sequence conservation. Second, independent to identified sequence families, we searched for conserved structure motifs using sequence-and-structure alignments. Structure motif candidates were constrained to be reminiscent of those previously published (17,19,20,22–25). More specifically, 33 small hairpin (or stem-loop) motifs with at least 4 bp and no bulges were identified. Their sequence conservation was generally lower than for sequence families: mean pairwise sequence identities between 47 and 94% with an average of 69% (compared with 82%). Sequence families and structure motifs were numbered according to size, starting with the largest clusters; the smallest cluster size was 10. Summary tables with sequence logos for families, secondary structures for motifs, mappings between families and motifs and annotations are available in the Supplementary Material; full alignments are available on the CRISPRmap web server.

To provide further support for our secondary structure predictions, we evaluated the motifs using the general ncRNA predictor, RNAz (51). Although RNAz is not specifically trained for CRISPR elements, it classified 79% (26 out of 33) of our motifs as structured ncRNAs with an SVM-RNA-class probability >0.6 (22 motifs even achieved >0.9, a clear indication that these motifs are evolutionary conserved). Compared with other ncRNA classes, RNAz only exhibits such promising sensitivities on some of the classical ncRNAs (52,53), for example, transfer RNAs or microRNAs, which are known for their distinct and well-defined secondary structures (54,55).

In total, out of all CRISPRs in our REPEATS data set, 64% were assigned to a conserved sequence family and 51% were assigned to a structure motif; 26% of repeats remained unassigned to either a family or motif, i.e. showed no conservation with available CRISPRs.

### A detailed visual map of CRISPR conservation

**Figure 1.**
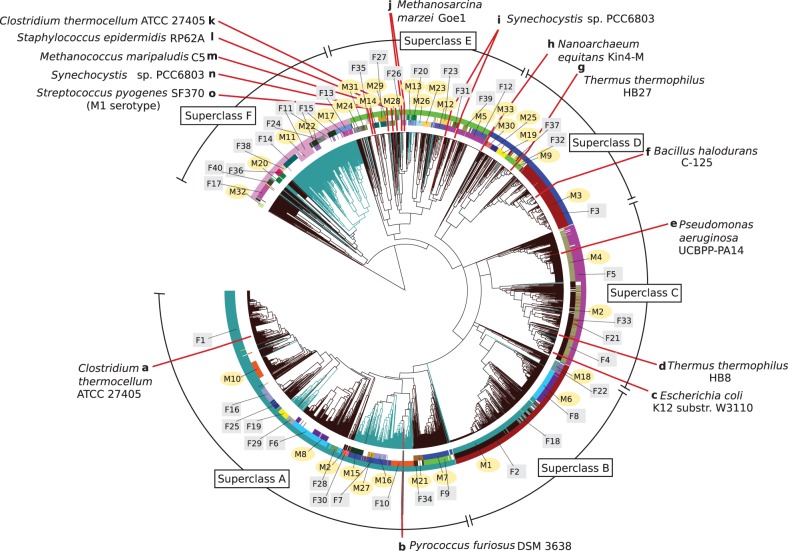
The CRISPRmap tree: a map of repeat sequence and structure conservation. The hierarchical tree is generated with respect to repeat sequence and structure pairwise similarity and the branches are coloured according to their occurrence in the domains bacteria (dark brown) or archaea (blue-green). The rings annotate the conserved structure motifs (inner), sequence families (middle) and the superclass (outer). Motifs and families are marked and highlighted with yellow circles, and grey squares, respectively. Finally, we marked locations of published CRISPR-Cas systems for which experimental evidence of the processing mechanism exists (13,17–25,33–36,51). A summary for these published systems is given in Supplementary Table S20. Repeats that show no conservation, i.e. were not assigned to either a sequence family or structure motif, were removed to clarify the visualisation.

As a visual *map* of both bacterial and archaeal CRISPR domains, we combined our categorisation into repeat families and motifs with a hierarchical tree based on sequence-and-structure similarities (see non-hierarchical, sequence-similarity-based visualisation in Supplementary Figure S9). This CRISPRmap tree details relationships between individual repeats and whole families and motifs (Figure 1).

In addition to the repeat families and motifs, we annotated taxonomic phyla, Cas1 sequence homology clusters, and Cas subtype annotations (8,10); the branches are coloured according to whether the CRISPRs stem from bacteria or archaea. We show one possible view of the CRISPRmap tree with structure-motifs, sequence-families and superclass classifications and the domain in Figure 1. Further views and annotation data are available in the supplementary material and on our CRISPRmap web server.

In summary, the CRISPRmap tree was designed to provide a visual overview of CRISPR conservation and to aid in the understanding of CRISPR-Cas diversity.

### The CRISPRmap tree is divided into six superclasses

**Figure 2.**
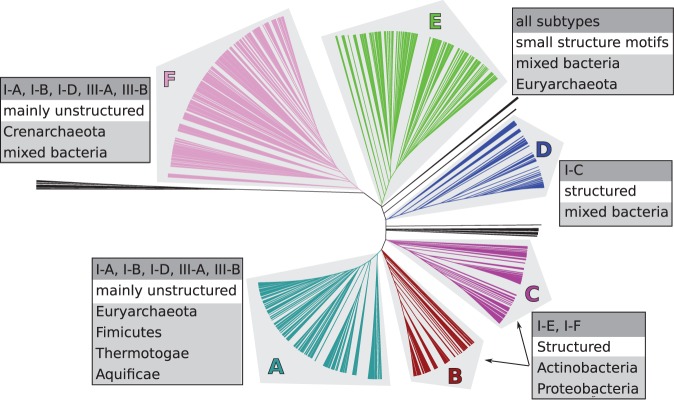
CRISPRs cluster into six major superclasses according to sequence and structure similarity. We summarised general results of our structure motif detection (i.e. structured or unstructured), Cas-subtype annotations (10) and taxonomic phyla beside each superclass.

Based on sequence-and-structure similarities and the tree topology, the REPEATS data set could be broadly grouped into six major superclasses (Figure 2). The superclasses, labelled A–F, are ordered according to generally decreasing conservation. The following information is quickly observed in the CRISPRmap tree (Figure 1): Superclass A contains highly conserved CRISPRs on the sequence level, but only a few small structure motifs. Superclasses B–C contain sequence families that roughly correspond to one structure motif each; the same is true for half of superclass D. The other half of superclass D and superclass E contain little sequence conservation, but many small conserved motifs. Archaeal CRISPRs in both superclasses A and F contain well-conserved sequence families and we find motifs for about half; however, these are less stable than the bacterial motifs in superclasses B–D (Supplementary Tables S2–S19). The bacterial repeats in superclass F are divergent. We included arrays with at least three repeat instances to ensure that our data set was complete. Many arrays with up to five repeat instances, however, show little conservation (Supplementary Figure S8): roughly 50% were not assigned to sequence families or structure motifs and most are in this diverse part of superclass F. In addition to array size, we marked repeats or spacers with unusual lengths on the CRISPRmap tree in Supplementary Figure S8. Some of the really short arrays, especially those with unusual repeat and/or spacer lengths are unlikely to contain functional CRISPRs.

We summarised subsequent annotations and clustering results to give a brief overview of each superclass in Figure 2; more details are given in the following results. In the CRISPRmap tree views (e.g., Figure 1), the superclass is always annotated in the outermost ring.

### Structure motifs fit to known cleavage sites

Most sequence families and structure motifs are associated with either bacterial or archaeal CRISPRs: only four motifs (M11, M20, M29 and M31) and one family (F20) contain a significant mixture of both domains. Bacterial CRISPRs are more structured in general than those from archaea. Although structured motifs were identified for both domains, the longer, more thermodynamically stable hairpins—associated with Cas subtypes I-C, I-E and I-F—belonged almost exclusively to bacterial CRISPRs in superclasses B–D (Supplementary Figure S10A–C and Supplementary Tables S6–S11). To add to the stability of such short hairpin motifs, 65% of base pairs are *G*s paired to *C*s. In a closer inspection, we observed that 94% of *GC* base pairs were orientated with the *G* toward the 3′-end (Supplementary Tables S2–S19). Such consecutive 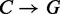 base pairs form a 3′ *G* side to the stem, which might be important for crRNA processing due to sequence specificity in this region (20,22,23).

In the literature, cleavage by known Cas6-like endoribonucleases (during crRNA maturation) occurs either at the 3′ base of the hairpin motif, or within the double-stranded region of the hairpin stem, usually below such a 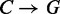 base pair (13,17–21,23–25,33–36). The product of this cleavage is an 8-nt-long repeat tag at the 5′-end of the mature crRNA (5′ tag), which corresponds to the last eight nucleotides from the 3′-end of the repeat sequence. Some exceptions to the 8-nt length exist (23,24,35,56,57). We located potential cleavage sites on our structure motifs according to published observations (17–20,22–25). Of all 33 structure motifs, 11 contain a potential cleavage site within the conserved stem of the motif of which 7 are below a 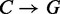 base pair. Another 13 motifs have a potential cleavage site at the 3′ base of the conserved stem. In Figure 2, we see that Cas subtypes I-E and I-F are split across the two superclasses B and C. This split is due to exactly one repeat-structure feature: the hairpin motifs are closer to the 3′-end of the CRISPRs in superclass B, resulting in a cleavage site within the stem. In superclass C, the cleavage site is at the base of the hairpin motif. In accordance to previously mentioned literature, the cleavage sites are below a 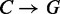 base pair in both superclasses. Aside from this difference in position, the hairpin structures associated with either I-E or I-F are similar.

### Sequence families exhibit variations in conservation

**Figure 3.**
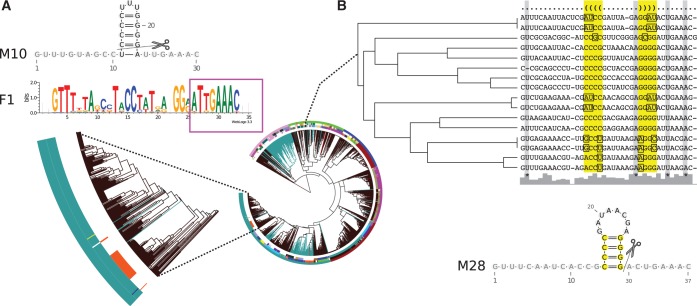
Highlighting the advantage of independent clustering approaches. (**A**) CRISPRs in the largest sequence family, F1, are mostly unstructured; however, for 50 CRISPRs also a conserved structure motif, M10, was identified. This indicates that subsets of conserved families can be structured. F1 contains the conserved 5′ tag, marked with the magenta box. (**B**) Structure motif M28 shows no sequence conservation, but a conserved structure (base pairs are highlighted in yellow). The many compensatory base pairs are marked in the alignment with squares. This structure has been verified via mutational analyses in (20). Potential cleavage sites are indicated as observed in the literature (13,17–21,23–25,33–36).

In a closer inspection of the family sequence logos, we see different patterns of *sequence* conservation (Supplementary Figure S10 and Supplementary Tables S2–S19). We highlight these difference using four selected examples: First, CRISPRs associated with the I-E subtype show a high conservation of *G*s and *C*s that form the base pairs of the hairpin motif. Second, CRISPRs associated with the I-F subtype are well-conserved across the entire repeat sequence and contain fewer consecutive *C*s and *G*s (Supplementary Figure S10A and B). Third, CRISPRs associated with the I-C subtype show a higher conservation at the base of the hairpin stem and in the single-stranded 5′- and 3′-ends, which suggests that the top of the stem and the hairpin loop is likely insignificant for the binding affinity (Supplementary Figure S10C); this conservation pattern is well-supported by mutation experiments in the type I-C system in *Bacillus halodurans* C-125 where crRNAs were still processed with a truncated upper stem and mutated hairpin loop, but processing was sequestered by mutations at the base of the stem or by the removal of the unpaired 3′-end (23). Fourth, in Figure 3, we marked the well-conserved 8-nt-long 5′ tag, 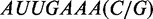, at the 3′-end of the repeats. Out of our 40 sequence families, 17 (∼40%) show a conservation of exactly this sequence tag; others contain minor deviations. Interestingly, bacterial superclasses B and C do not show this tag, whereas it is highly conserved throughout the other bacterial superclass D and in almost all archaeal families (9 out of 12). We hypothesise that these patterns of conservation give a good indication of differences in binding affinities for specific Cas proteins in the various CRISPR-Cas systems.

### Sequence families and structure motifs provide independent information about evolution

Structured ncRNA families cannot be identified by sequence conservation alone because standard alignment tools fail when the pairwise sequence identity is <60% (58). We see the same tendency for structured and unstructured repeats in our data: The CRISPRmap tree shows different patterns of overlap between sequence families and structure motifs that we identified by independent clustering approaches (Figure 1). In Figure 3, we highlight two overlap patterns. First, in superclass A, the largest family, namely F1, is mainly unstructured. For a subset of these CRISPRs, however, we identified a thermodynamically stable hairpin motif (M10) with four consecutive 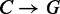 base pairs; these CRISPRs are clearly structured. Second, in superclass D, we found a conserved hairpin motif (M28), also with four consecutive 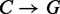 base pairs and a large 8-nt hairpin loop that was verified by mutational analyses in a type III-A system in *Staphylococcus epidermidis* RP62A (20); this motif does not show enough sequence conservation to be detected as a sequence family. Both M10 and M28 would not have been identified with the approach used in (32), in which consensus structures were calculated from (entire) sequence families. In addition, we observe cases where a structure motif corresponds almost fully to a sequence family, e.g. M1 with F2 and M2 with F4. Nevertheless, individual members of the sequence families cannot form the associated consensus structure: this may indicate a degenerate and non-functional CRISPR-Cas system, or one that has evolved to function with a different or no repeat structure.

### A subset of Cas subtypes are weakly linked to repeat and Cas1 evolution

**Figure 4.**
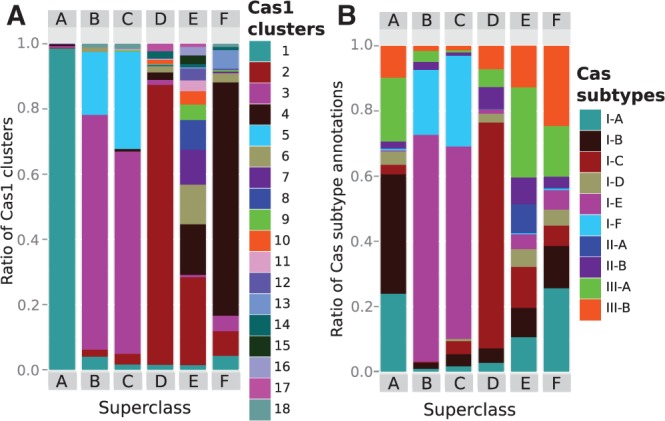
Relative ratios of Cas1 sequence clusters and Cas-subtype annotations per superclass. (**A**) Cas1 sequence clusters correspond well to the superclass and thus the CRISPRmap tree with the exception of superclass E; superclass E is diverse in both repeat and associated Cas1 conservation and it probably contains only partial data. (**B**) Bacterial CRISPRs that are assigned to well-defined structure motifs are associated with subtypes I-C, I-E and I-F in superclasses B–D and are strongly linked to both repeat and Cas1-sequence similarities (i.e. CRISPR evolution). Superclass A and F contain both bacterial and archaeal CRISPRs (many are unstructured), which are loosely associated with the remaining type I and both type III subtypes. These subtypes do not correspond to Cas1 and repeat evolution and are likely composed of interchangeable protein complexes or modules. The diversity of superclass E is also reflected by the mixture of all subtypes; in addition, the majority of type II CRISPRs are also located in this region.

From the literature, we already know that Cas1 is strongly linked to repeat evolution (12,59). This link could be verified for our large-scale data set (Figure 4A). We clustered associated Cas1-protein sequences and the resulting Cas1 clusters fit well with the superclasses, except superclass E (Figure 4). There are several indications that superclass E contains only partial data, e.g. conserved sequence families and structure motifs are smaller and most CRISPRs show little to no conservation; however, 50% of the CRISPRs from metagenomic data in the subsequent use-case study fall into this superclass and new conserved classes are indicated (Supplementary Figure S7).

For Cas-subtypes (10), the linkage pattern is different: subtypes I-C, I-E and I-F correlate well with repeat (and thus Cas1) conservation, whereas the remaining type I and both type III Cas subtypes are only weakly linked (Figure 4). The bacterial superclasses B, C and D contain well-defined structure motifs and sequence families (Figure 1 and Supplementary Tables S2–S19), which are associated with subtypes I-E and I-F (superclasses B and C) and I-C (half of superclass D). Superclasses A and F contain both bacterial and archaeal CRISPRs—most of which are unstructured—and although they also fit well to the Cas1 clusters, the annotated Cas subtypes are a diverse mixture of the remaining type I subtypes (I-A, I-B and I-D) and both type III subtypes (Figure 4). In superclass E, we observe a similar co-occurrence of these subtypes; however, this superclass contains all subtypes owing to aforementioned diversity and incomplete data.

There are two possible explanations for the co-occurrence of type I and type III subtypes. First, these subtypes are composed of interchangeable modules as previously mentioned for archaeal systems in (12,60). In such cases, one would expect Cas proteins from different subtypes to be able to process similar repeat sequences; two examples in the literature that support this theory is a Cas6 (Cas6b) protein that can process both type I-B systems in *Methanococcus maripaludis* C5 and *Clostridium thermocellum* ATCC 27405 (13) and two CRISPRs in *Methanosarcina marzei* Gö1 with near-identical repeats are associated with different subtypes I-B and III-B (25). Also, many sequence families and structure motifs co-occur with multiple, or a mixture of, subtypes (see Supplementary Tables S2–S19 and web server). The co-occurrence of subtypes is widespread in archaea and bacteria. In general, an exchange of protein modules would require compatible repeat sequences and structures. The only similarity observed in CRISPRs associated with mixed subtypes is the conserved 5′ tag—*AUUGAAA(C/G)*—or a slight variation. In comparison, repeats associated with the bacterial subtypes I-E and I-F do not contain this tag. Second, additional or unknown Cas proteins are required to achieve a subclassification of Cas subtypes that is more compatible with repeat conservation. Most likely, the truth lies in a combination of both explanations. Finally, subtypes I-A, I-B, I-D, III-A and III-B are more enriched in extremophiles, e.g. thermophiles (Supplementary Figure S6).

### CRISPRs in Euryarchaeota are closer to bacterial systems than ones in Crenarchaeota

Ninety-seven precent of the archaeal CRISPRs originate from two phyla: 380 from Euryarchaeota and 245 from Crenarchaeota. In the CRISPRmap tree (Figure 1 and Supplementary Figure S4), we observe a clear separation of these two CRISPR groups: 60% of CRISPRs from Euryarchaeota and 96% from Crenarchaeota cluster into superclasses A and F, respectively. In superclass A, the euryarchaeal and bacterial CRISPRs are associated with Cas1 proteins that cluster into the same Cas1-cluster-1, i.e. these Cas1 sequences are evolutionarily close (Figure 4). In contrast, CRISPRs from Crenarchaeota are located almost exclusively in a subregion of superclass F and are associated with the separate Cas1-cluster-4 (Supplementary Figure S4).

### Evidence of horizontal transfer

As previously mentioned, archaeal and bacterial CRISPRs are distinctly separated in the CRISPRmap tree (Figure 1). This is consistent with a rare exchange of genetic material between archaeal and bacterial systems (11,12). Nevertheless, we observed a few instances where archaeal repeats are located in a bacterial-dominated region and vice versa (see Supplementary Methods S1.4 for more details). With one exception, all cases involved a transfer of the CRISPR-Cas system from bacteria to archaea; archaea have also been shown to uptake bacterial and eukaryotic DNA as spacers (61). Supplementary Figure S11 gives examples of archaea that contain full bacterial CRISPR-Cas systems where a strong conservation of the structure motif is supported by multiple compensatory base pair mutations. In addition, not only the Cas1 proteins are conserved, but the archaeal CRISPRs are associated with the complete set of proteins from the bacterial subtypes I-C and I-E.

The transfer of genetic material between prokaryotes often occurs via plasmids; however, in Supplementary Figure S11, all horizontally transferred systems are located on chromosomes and not on plasmids. In fact, only 7% of over 1300 plasmids analysed contained a CRISPR array. Therefore, it is unlikely that the dominant mechanism of transferring CRISPR-Cas systems between organisms is via plasmids.

### The CRISPRmap web server

The CRISPRmap web server enables easy access to our data and allows scientists to compare the conservation of individual repeats. Repeats are entered in FASTA format and the web server automatically assigns them to our classification system; previously unknown repeats are assigned to existing families and/or motifs, if possible. Non-conserved input sequences remain unassigned, but are still located according to their relative similarity in the tree. Furthermore, if the correct orientation of the input repeats is unknown, the user can request to predict the orientations to ensure that they are consistent with our data.

#### A use-case study

A valuable source of new CRISPR-Cas systems are metagenomic studies of multiple, often novel, prokaryotic organisms. Recently, a targeted search for CRISPR arrays was performed in the bacterial metagenome of different sites on the human body (62). In this study, 150 CRISPRs were identified that could potentially be used to learn more about invader patterns. We applied the CRISPRmap web server to determine the conservation of these CRISPRs at a quick glance: only 38 and 29% were assigned to our structure motifs or sequence families, respectively. Notably, 50% of the metagenomic CRISPRs were assigned to the diverse superclass E where most remained unassigned to either a structure motif or sequence family; however, in Supplementary Figure S7, many of these repeats cluster together to potentially form new classes of motifs and families. Two CRISPRs fall into the euryarchaeal region in superclass A, despite the fact that archaea are rarely associated with human microbiomes (62). These results highlight the fact that even with the large-scale analysis performed in this work, we still do not know the full extent of CRISPR-Cas diversity. Therefore, the dynamic nature of our web server—in the fact that it allows the classification of newly sequenced CRISPRs to be assigned to existing sequence families and structure motifs—is particularly useful.

## CONCLUSION

We provide a comprehensive analysis of CRISPR structure and sequence conservation based on the largest data set of repeat sequences available. We show extensively that our methods are well suited to identifying many characteristics of CRISPR-Cas systems: e.g. cleavage sites, patterns of RNA structure motifs and sequence conservation, the link between evolution of CRISPRs and associated Cas subtypes and the horizontal transfer of such systems. On the one hand, specific conservation patterns can be combined with published data to make assumptions about CRISPRs belonging to the same sequence families or structure motifs. On the other hand, the CRISPRmap overview can be used to find potentially novel CRISPR-Cas systems that are highly divergent from the rest. User-based queries on our data enable more informed choices on future hypotheses in CRISPR-Cas research.

## SUPPLEMENTARY DATA

Supplementary Data are available at NAR Online, including [63,64].

Supplementary Data
