# Supplementary material for: CRISPRmap: an automated classification of repeat conservation in prokaryotic adaptive immune systems
Source: Nucleic Acids Res. 2013 Jul 17;41(17):8034–44. doi: 10.1093/nar/gkt606 (PMC3783184; doi:10.1093/nar/gkt606)
Supplement: Supplementary Data [file supp_41_17_8034__index.html]

CRISPRmap: an automated classification of repeat conservation in prokaryotic adaptive immune systems — CRISPRmap: an automated classification of repeat conservation in prokaryotic adaptive immune systems — Supplementary Data 

# CRISPRmap: an automated classification of repeat conservation in prokaryotic adaptive immune systems

## 

files

**Files in this Data Supplement:**

- Supplementary Data - pdf file
