# Supplementary material for: CRISPRmap: an automated classification of repeat conservation in prokaryotic adaptive immune systems
Source: Nucleic Acids Res. 2013 Jul 17;41(17):8034–44. doi: 10.1093/nar/gkt606 (PMC3783184; doi:10.1093/nar/gkt606)
Supplement: Supplementary Data [file supp_gkt606_nar-01318-z-2013-File001.pdf]

Supplementary information
CRISPRmap: an automated classification of repeat conservation in prokaryotic adaptive immune systems

Sita J. Lange[1,#] , Omer S. Alkhnbashi[1,#], Dominic Rose[1,#], Sebastian Will[1,5]and Rolf Backofen[1,2,3,4,*]

[1]Bioinformatics Group, Department of Computer Science, Albert-Ludwigs-Universität Freiburg, Georges-Köhler-Allee 106, 79110 Freiburg, Germany
[2]ZBSA Centre for Biological Systems Analysis, University of Freiburg, Habsburgerstr. 49, 79104 Freiburg, Germany
[3]BIOSS Centre for Biological Signalling Studies, Cluster of Excellence, University of Freiburg, Germany
[4]Center for non-coding RNA in Technology and Health, University of Copenhagen, Gronnegardsvej 3, DK-1870 Frederiksberg C, Denmark
[5]Bioinformatics, Department of Computer Science, University of Leipzig,04107 Leipzig, Germany
[#]These authors contributed equally to this work

Email: Sita J. Lange - sita@informatik.uni-freiburg.de; Omer S. Alkhnbashi - alkhanbo@informatik.uni-freiburg.de; Dominic Rose - dominic@informatik.uni-freiburg.de; Sebastian Will - will@bioinf.uni-leipzig.de; Rolf Backofen*- backofen@informatik.uni-freiburg.de;

[*]Corresponding author

S1 Additional methods

S1.1 Cas subtype annotation from Haft et al. 2005.

To annotate the early Cas subtypes from Haft *et al.* [1], we followed the procedure given in Kunin *et al.* [2]. More specifically, we downloaded the single *cas* gene models created by Haft *et al.* from the TIGRFAM database. Using the HMMER program with the TIGRFAM models (same as for the single *cas* gene annotation), we searched the 20 kb of nucleotides up- and downstream of the array locus and annotated a *cas* gene if it was found with an E-value ≤ 0.001. We used a strict annotation of Cas subtypes, whereby all *cas* genes of a subtype were required.

S1.2 Webserver input: adding new repeat sequences to the existing CRISPR clustering

The user of our CRISPRmap webserver can enter any CRISPR sequences and they will be assigned to our sequence families and structure motifs, if possible, and integrated into the hierarchical CRISPRmap tree. Thus, information on conservation is available for not only sequences in our dataset, but also novel, yet unsequenced, CRISPRs. In the following, we describe the procedure for one input sequence, many sequences are done simultaneously in the same way:

1. *Is the repeat sequence in our database?* If the given repeat sequence is in our database, in either

orientation, we highlight this sequence (or one if many copies exist) in our CRISPRmap cluster tree, and automatically assign it to the corresponding structure motif and/or sequence family and stop here.

2. *What is the correct orientation?* If the user is not sure about the correct repeat orientation, i.e. the checkbox for repeat orientation has been activated, we first predict the orientation with our model described in the methods section of the main manuscript. The orientation should then be consistent with our data.

3. *Is it structured or unstructured?* The RNA structure prediction algorithm, RNAfold [3] is used to determine whether the repeat sequence is structured or unstructured. If the minimum free energy structure is the unstructured sequence, i.e. contains no base-pairs, it remains unassigned to a structure motif and we continue with Step 5.

4. *Does it belong to a structure motif?* Albeit a structure being predicted, the repeat does not necessarily belong to a conserved structure motif. We add the repeat sequence to all repeats assigned to one of our structure motifs and re-run RNAclust [4] with a modified UPGMA algorithm (see following section "Constrained Clustering"). In short, the modification allows the generation of the cluster tree by keeping the motifs intact, i.e. non-overlapping. If a repeat falls into or next to one of the existing structure motifs, we assign it to the motif by the following: (1) The repeat is folded by RNAfold [3] with the option -p to calculate a structure dotplot. (2) This dotplot is aligned with the consensus dotplot of the structure motif using LocARNA. (3) The repeat is assigned to be a member of the motif if it is able to fold into the consensus structure of that respective motif with at most one base-pair missing. We ensure that the new consensus structure contains at least four base-pairs and is at the same position as previously. A comparison of the new and old consensus structures and alignments is given on the web server results page.

5. *Does it belong to one of our conserved sequence families?* We assign the repeat to a conserved sequence family by comparing it to the previously calculated ClustalW sequence profiles [5], see Methods section "Clustering of repeat sequences into conserved sequence families". Let $sim(F, r)$ be the profile score of a repeat r compared with the profile of the family F, where $r \notin F$. For each family, the minimum F_{min} and maximum F_{max} profile similarity was determined by removing each sequence from the family, re-calculating the profile for the remaining sequences, and determining the similarity score of the respective repeat to the profile. A repeat r was then assigned to a sequence family F if (1) $sim(F, r)$ is greater or equal to F_{min} and (2) the distance between $sim(F, r)$ and F_{max} is the minimum for all families.

6. *Where is it located in the CRISPRmap cluster tree?* With a final run of RNAclust on all repeat sequences, we get the updated CRISPRmap cluster tree and we highlight the input sequence location in this tree. Any additional annotations (outer rings), such as Cas subtype, are not displayed for novel repeat sequences.

S1.3 Constrained Clustering

We consider the general problem to cluster a set of taxa hierarchically based on their distances. Additionally, we constrain the clustering such that certain, e.g. a priori known, clusters are prevented from mixing with each other.

Given is a set of taxa, indexed from 1 to n, together with all pairwise distances between the taxa; furthermore, a set $\mathcal{X}$ of disjoint clusters of these taxa, i.e. $\mathcal{X}$ is contained in the powerset of $\{1, \dots, n\}$ and all non-identical clusters c and d in $\mathcal{X}$ do not intersect. Commonly, $\mathcal{X}$ covers only a subset of all taxa;

therefore, we distinguish *constrained taxa* (that are contained in some element of $\mathcal{X}$) and the remaining *unconstrained taxa.*

We aim to construct a cluster tree of the taxa, i.e. a rooted binary tree T with n leaves corresponding to the n taxa. First, this tree should reflect the given distances. Second it has to support the clustering given by $\mathcal{X}$ such that clusters in $\mathcal{X}$ are grouped together but unconstrained taxa can be interspersed freely. For this purpose, we require that no subtree of T contains leaves from two different clusters in $\mathcal{X}$ unless both clusters are completely contained in the subtree. We call this condition *$\mathcal{X}$-cluster constraint.* (Formally: for each subtree with leaves L and each pair of non-identical clusters c and d in $\mathcal{X}$, $c \cap L \subset c$ implies $d \cap L = \emptyset$.)

Our novel constrained clustering algorithm is based on the unweighted pair group method UPGMA. The original algorithm UPGMA starts from n singleton clusters corresponding to the n taxa. Until all clusters are combined, it iteratively merges the two nearest clusters. For the latter, the cluster distances are initially derived from the input distances and distances to new clusters are computed after each merge of clusters. The sequence of merges determines the cluster tree. The novel algorithm modifies UPGMA, such that, in each iteration, it merges the nearest pair of clusters that can be merged without violating the $\mathcal{X}$-cluster constraint. To check this condition efficiently, we keep track for each cluster whether it contains some elements of a cluster in $\mathcal{X}$ and whether it includes such a cluster completely. Merging two clusters does violate the constraint if and only if each cluster overlaps some cluster in $\mathcal{X}$ but does not cover it completely.

S1.4 Horizontal gene transfer between bacteria and archaea

Although archaeal CRISPRs are generally well-separated from bacterial ones in general, we observed a few instances where an archaeal CRISPR is located within a bacterial-dominated region and vice versa. To investigate whether these mixed regions could arise from potential horizontal transfer, we applied BLAST to search for homologous Cas1 (or Cas2) protein sequences (Cas1 and Cas2 are the most ubiquitous Cas proteins and exist in both bacteria and archaea). We identified 24 archaeal and 8 bacterial repeats that were assigned to sequence families or structure motifs dominated by the opposite domain. For 75% (18 out of 24) of the archaeal repeats, we identified Cas1 or Cas2 homologs in bacteria in the top five BLAST hits (E-value $\leq 2 \times 10^{-10}$); the same was true for only one of the four bacterial repeats.

S2 Supplementary tables

S2.1 Number of Cas subtype annotations

We annotated each CRISPR in our dataset according to the closest Cas subtypes as described in the methods of the manuscript. The two major Cas subtype annotation systems were considered [1, 6]; the number of CRISPRs we annotated with each subtype is given in Table S1.

S2.2 Summary tables of sequence families and structure motifs

Supplementary Tables S2–S19 summarise the sequence families and structure motifs, sorted according to the superclass they belong to. The numbering of the families is according to the number of repeats belonging to that family. The annotations in each column is done manually with respect to the majority of repeats in that family (see other supplementary file for the full list). For the Cas subtype, an annotation is

Subtype	Archaea	Bacteria	Total
10 subtypes from Makarova *et al.* 2011 [6]			
I-A	134	203	337
I-B	89	293	382
**I-C**	**14**	**322**	**336**
I-D	49	38	87
**I-E**	**8**	**447**	**455**
**I-F**	**1**	**155**	**156**
II-A	0	50	50
II-B	9	95	104
III-A	148	223	371
III-B	108	149	257
% CRISPR	87 %	68 %	72 %
8 subtypes from Haft *et al.* 2005 [1]			
Apern	65	0	65
**Dvulg**	**1**	**184**	**185**
**Ecoli**	**8**	**369**	**377**
Hmari	15	36	51
Mtube	8	9	17
Nmeni	0	27	27
Tneap	89	254	343
**Ypest**	**0**	**120**	**120**
% CRISPR	29 %	35 %	34 %

Table S1: The number of identified Cas subtype annotations for our REPEATS dataset. There were double as many annotations using the more recent classification from Makarova *et al.*, however, we did not require that all *cas* genes from the respective subtype to be present; whereas the annotations performed for Haft *et al.* were more strict, since we used full subtype models (see methods). In general, Dvulg, Ecoli, Hmari, Mtube, Nmeni, and Ypest correspond to I-C, I-E, I-B, III-A, both type II, and I-F, respectively. Structured repeats with very stable and conserved hairpin motifs, mainly found in bacteria, are written in bold. Note that the 9 subtype II-B CRISPRs in archaea are likely to be incorrect as we did not identify an RNase III in these organisms. Automated annotation of subtype II-B was especially difficult as it contains no subtype-specific Cas protein.

only given if this is more or less clear. If there is a complete mix of subtypes, no information is given. The Cas subtypes are summarised according to the *cas* genes that are found in the majority of chromosomes which contain the CRISPRs of each family or motif. More details of the majority *cas* genes is given on the web server. Archaeal families and motifs are highlighted in blue. If the CRISPRmap webserver is updated in future, then these tables supply a record for sequence families and structure motifs that are referred to in this work. The secondary structures of the motifs and sequence logos of the families are also provides in the tables.

Table S2: **Summary for the bacterial sequence families in Superclass A.**

#	Sequence Logo	Size	Motifs	Taxonomy	Subtypes
F1	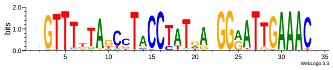	289	M10<br>un-structured	Firmicutes	I-B<br>III-A<br>III-B
F25	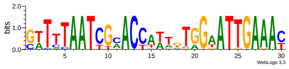	23	un-structured	mixed bacteria	I-A<br>II-B<br>III-A
F16	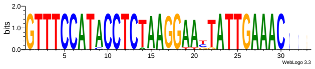	40	un-structured	Thermotogae	III-A
F30	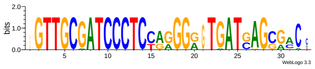	19	M2	Actinobacteria	-
F6	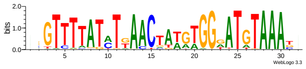	124	M8<br>un-structured	Firmicutes	I-A
F28	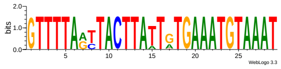	20	un-structured	Firmicutes	I-A
F34	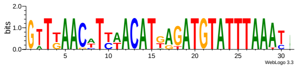	15	M21	Firmicutes	II-B
F9	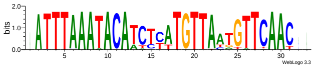	76	M7	Firmicutes	III-B

Table S3: **Structure motif summary for bacterial motifs in Superclass A.**

#	Structure Motif	Size	Families	Taxonomy	Subtypes
M10	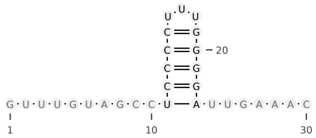	50	F1	Firmicutes	I-B<br>II-B<br>III-A
M8	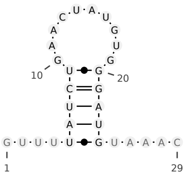	55	F6	Firmicutes	I-A<br>I-B<br>III-A
M21	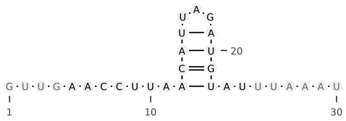	26	F34<br>unassigned	Firmicutes	-
M7	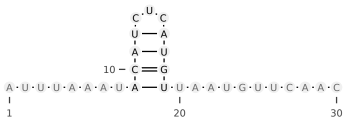	78	F9	Firmicutes	I-A<br>III-B

Table S4: **Summary for the archaeal sequence families in Superclass A.**

#	Sequence Logo	Size	Motifs	Taxonomy	Subtypes
F29	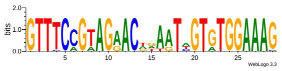	20	un-structured	Euryarchaeota Crenarchaeota	III-A
F19	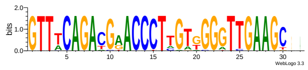	32	un-structured	Euryarchaeota	-
F7	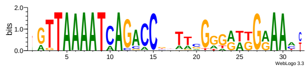	108	M15 M16 M27	Euryarchaeota	I-A
F10	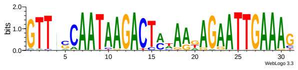	70	un-structured	Euryarchaeota	I-B

Table S5: **Structure motif summary for archaeal motifs in Superclass A.**

#	Structure Motif	Size	Families	Taxonomy	Subtypes
M15	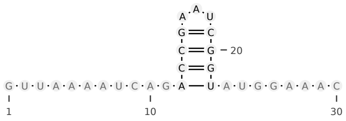	35	F7	Euryarchaeota	-
M27	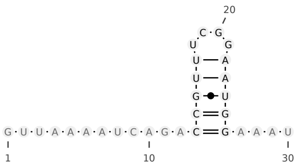	17	F7	Euryarchaeota	-
M16	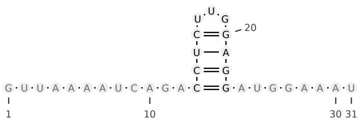	33	F7	Euryarchaeota	-

Table S6: **Sequence family summary for Superclass B.**

#	Sequence Logo	Size	Motifs	Taxonomy	Subtypes
F2	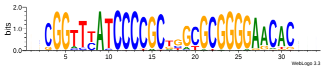	221	M1	Actinobacteria<br>Proteobacteria	I-E
F18	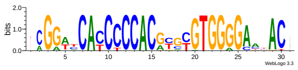	35	M1	mixed bacteria	I-E<br>II-B
F8	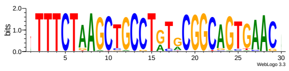	88	M6	Proteobacteria	I-F
F22	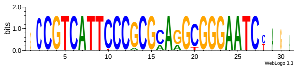	26	M18	Proteobacteria	I-E

Table S7: **Structure motif summary Superclass B.**

#	Structure Motif	Size	Families	Taxonomy	Subtypes
M1	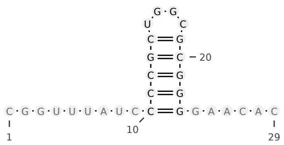	265	F2<br>F18	mixed bacteria	I-E
M6	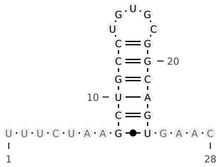	89	F8	Proteobacteria	I-F
M18	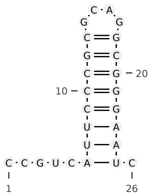	28	F22	Proteobacteria	III-B

Table S8: **Sequence family summary for Superclass C.**

#	Sequence Logo	Size	Motifs	Taxonomy	Subtypes
F4	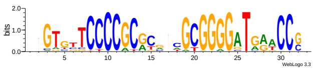	172	M2	Actinobacteria<br>Proteobacteria	I-C<br>I-E<br>II-B
F21	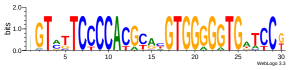	27	M2	mixed bacteria	I-E
F33	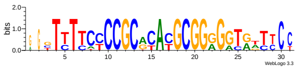	16	M2	mixed bacteria	I-C<br>I-E<br>II-B
F5	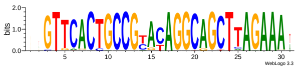	135	M4	Proteobacteria	I-F

Table S9: **Structure motif summary for Superclass C.**

#	Structure Motif	Size	Families	Taxonomy	Subtypes
M2	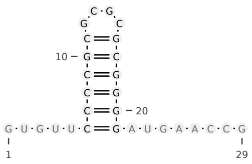	222	F4<br>F21<br>F30<br>F33<br>unassigned	mixed bacteria	I-E
M4	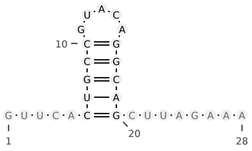	142	F5<br>unassigned	Proteobacteria	I-F

Table S10: **Sequence family summary for Superclass D.**

#	Sequence Logo	Size	Motifs	Taxonomy	Subtypes
F3	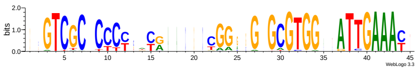	210	M3<br>M9	mixed bacteria	I-C
F37	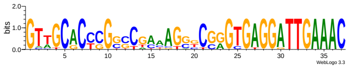	14	M9	Deinococcus-<br>Thermus	I-C<br>III-B
F32	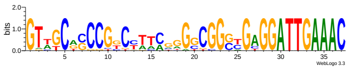	18	M9	Deinococcus-<br>Thermus<br>Proteobacteria	I-C

Table S11: **Summary for structure motifs in Superclass D with sequence conservation.**

#	Structure Motif	Size	Families	Taxonomy	Subtypes
M3	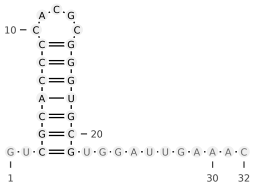	195	F3	mixed bacteria	I-C
M9	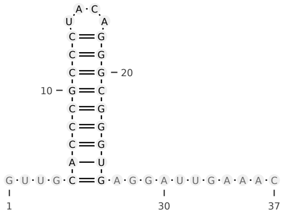	52	F3<br>F32<br>F37	mixed bacteria	I-C<br>I-A

Table S12: **Summary for structure motifs in Superclass D without sequence conservation.**

#	Structure Motif	Size	Families	Taxonomy	Subtypes
M19	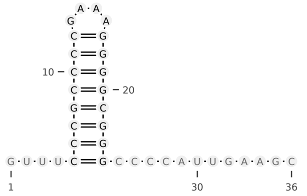	28	unassigned	mixed bacteria	I-A<br>II-B<br>III-B
M25	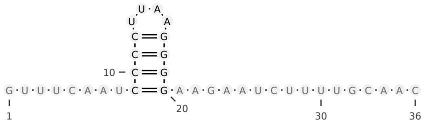	19	unassigned	mixed bacteria	III-A<br>III-B
M30	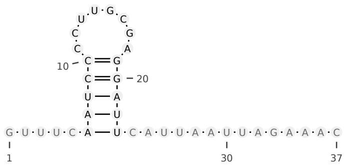	13	unassigned	Cyanobacteria<br>Chloroflexi	I-E<br>II-B
M33	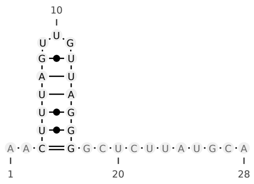	10	unassigned	mixed bacteria	II-B

Table S13: **Sequence family summary for Superclass E.**

#	Sequence Logo	Size	Motifs	Taxonomy	Subtypes
F39	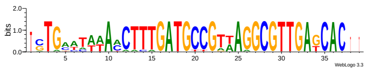	13	M5	mixed bacteria	I-A<br>I-B<br>II-B
F31	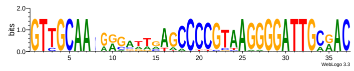	19	M5	Deinococcus-Thermus	III-A
F12	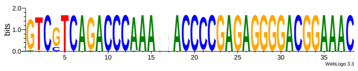	45	M5	Actinobacteria	II-B<br>III-A
F23	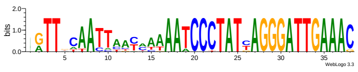	24	M12	Cyanobacteria	I-D<br>II-B
F20	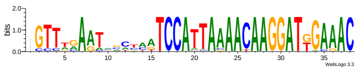	28	M13<br>un-structured	Euryarchaeota<br>mixed bacteria	I-B
F26	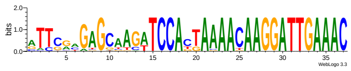	23	M13<br>un-structured	Euryarchaeota	-
F35	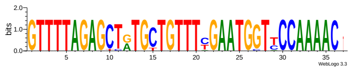	15	un-structured	Firmicutes	II-A
F27	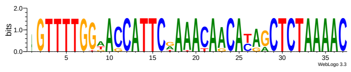	22	M14<br>un-structured	Firmicutes	II-A<br>II-B

Table S14: **Summary of bacterial structure motifs in Superclass E with sequence conservation.**

#	Structure Motif	Size	Families	Taxonomy	Subtypes
M5	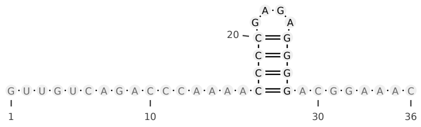	106	F12<br>F31<br>F39<br>unassigned	Cyanobacteria<br>mixed bacteria	II-B<br>III-A
M12	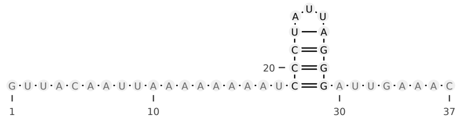	40	F23<br>unassigned	mixed bacteria	-
M14	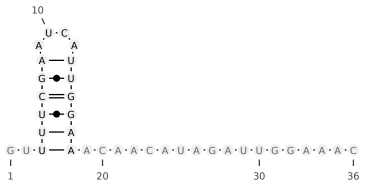	35	F27<br>unassigned	Firmicutes<br>Cyanobacteria	II-A<br>II-B

Table S15: **Summary of bacterial structure motifs in Superclass E without sequence conservation.**

#	Structure Motif	Size	Families	Taxonomy	Subtypes
M23	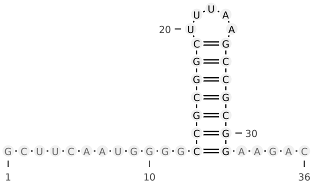	23	unassigned	mixed bacteria	-
M26	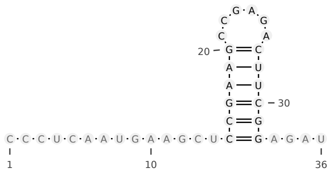	19	unassigned	Actinobacteria	-
M28	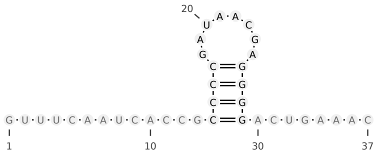	16	unassigned	mixed bacteria	I-C<br>III-A
M24	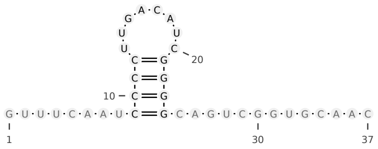	21	unassigned	mixed bacteria	-

Table S16: **Summary of archaeal structure motifs in Superclass E.**

#	Structure Motif	Size	Families	Taxonomy	Subtypes
M13	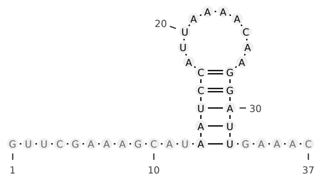	37	F20<br>F26	Euryarchaeota	I-A
M31	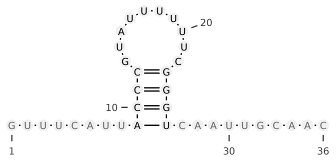	11	unassigned	Euryarchaeota<br>mixed bacteria	-
M29	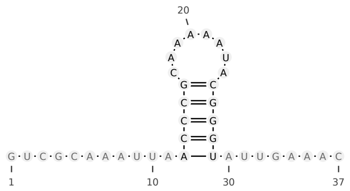	14	unassigned	Euryarchaeota<br>mixed bacteria	II-B

Table S17: **Sequence family summary for Superclass F.**

#	Sequence Logo	Size	Motifs	Taxonomy	Subtypes
F24	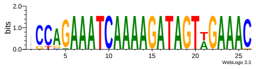	23	un-structured	Crenarchaeota	III-A<br>III-B
F15	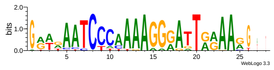	42	M22<br>un-structured	Crenarchaeota	I-A<br>III-B
F13	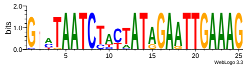	44	M17<br>un-structured	Crenarchaeota	I-A<br>III-B
F11	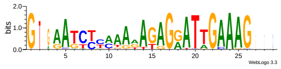	49	M11<br>un-structured	Crenarchaeota	III-B
F14	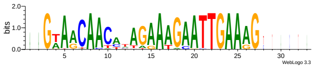	44	un-structured	Crenarchaeota	I-A<br>I-D<br>III-A
F38	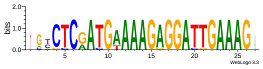	13	un-structured	mixed archaea	I-A<br>III-B
F36	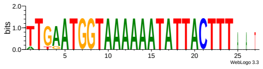	15	M20	Firmicutes	-
F40	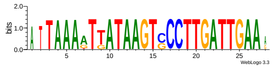	13	un-structured	Proteobacteria	I-B
F17	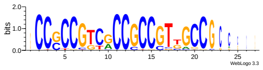	39	un-structured	Actinobacteria	-

Table S18: **Summary for archaeal structure motifs in Superclass F.**

#	Structure Motif	Size	Families	Taxonomy	Subtypes
M22	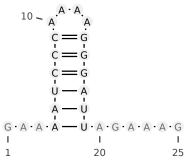	24	F15	Crenarchaeota	I-A<br>III-B
M17	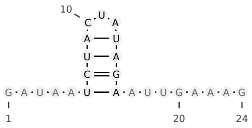	29	F13	Crenarchaeota	I-A<br>III-B
M11	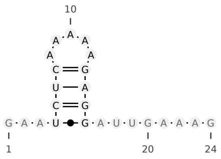	45	F11<br>unassigned	Crenarchaeota	III-A<br>III-B
M20	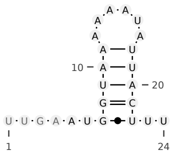	27	F36<br>unassigned	Firmicutes<br>Crenarchaeota	-

Table S19: **Final structure motif unassigned to a Superclass.**

#	Structure Motif	Size	Families	Taxonomy	Subtypes
M32	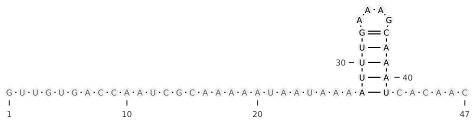	10	unassigned	Becteroidetes	II-B

Table S20: Published CRISPR-Cas systems with experimental evidence of the processing mechanism. In particular, these are systems for which the Cas endoribonuclease is characterised and/or the repeat structure has been verified. Published results are consistent with our data. The IDs, a–o, are marked, in order, as red lines on the CRISPRmap tree in the manuscript in Figure 1.

ID	Organism	Family	Motif	Cas Subtype	Summary
**Superclass A**					
a	*Clostridium thermocellum* ATCC 27405	F1	-	I-B	Unstructured; 8-nt-5'-tag; biochemical evidence to show **Cas6b** activity [7]
b	*Pyrococcus furiosus* DSM 3638	F10	-	III-B	Unstructured; 8-nt-5'-tag; cleavage by **Cas6**; crystal structure of repeat wrapped around Cas6 [8]
**Superclass C**					
c	*Escherichia coli* K12 substr. W3110	F4	M2	I-E	Structure predicted, but stable; 8-nt-5'-tag; cleavage by **Cas6e**, biochemical experiments [9]
d	*Thermus thermophilus* HB8	F4	M2	I-E	Structured; 8-nt-5'-tag; cleavage by **Cas6e**; crystal structure of repeat hairpin in Cas6e (Cse3) [10, 11]
e	*Pseudomonas aeruginosa* UCBPP-PA14	F5	M4	I-F	Cleavage by Cas6f (Csy4); 8-nt-5'-tag; crystal structure and mutational analyses of repeat hairpin in **Cas6f** [12–14]
**Superclass D**					
f	*Bacillus halodurans* C-125	F3	M3	I-C	Cleavage by **Cas5d**; 11-nt-5'-tag mutational analysis of hairpin structure [15]
g	*Thermus thermophilus* HB27	F37	M9	I-C	Cleavage by **Cas5d**; 11-nt-5'-tag biochemical experiments [16]
h	*Nanoarchaeum equitans* Kin4-M	-	-	I-A	Biochemical evidence to show **Cas6b** activity; 8-nt-5'-tag [17]
**Superclass E**					
i	*Synechocystis* sp. PCC6803	-	M5	I-D & III-variant	Cleavage by **Cas6**; 8-nt-5'-tag; biochemical experiments, extended structure prediction of hairpin motif [18]
j	*Methanosarcina marzei* Gö1	F26	M13	I-B & III-B	Cleavage by **Cas6b**; 8-nt-5'-tag; structure probing experiment of hairpin [19]
k	*Clostridium thermocellum* ATCC 27405	F20	-	I-B	Biochemical evidence to show **Cas6b** activity; 8-nt-5'-tag [7]
l	*Staphylococcus epidermidis* RP62A	-	M28	III-A	Cleavage by **Cas6**; 8-nt-5'-tag; hairpin structure as in M28 verified by mutational analysis and sequence specificity around cleavage site [20]
m	*Methanococcus maripaludis* C5	-	M29	I-B	Cleavage by **Cas6b**; 8-nt-5'-tag; biochemical experiments [7]
n	*Synechocystis* sp. PCC6803	-	M14	III-variant	Biochemical analysis of **Cmr2** implicate its involvement in either cleavage, crRNA stabilisation, or array expression regulation; 13-nt-5'-tag [18]
o	*Streptococcus pyogenes* SF370 (M1 serotype)	F35	-	II-A	Cleavage with **tracrRNA**, host **RNase III** and **Cas9**, biochemical experiments; 22-nt-5'-tag [21]

S3 Supplementary figures

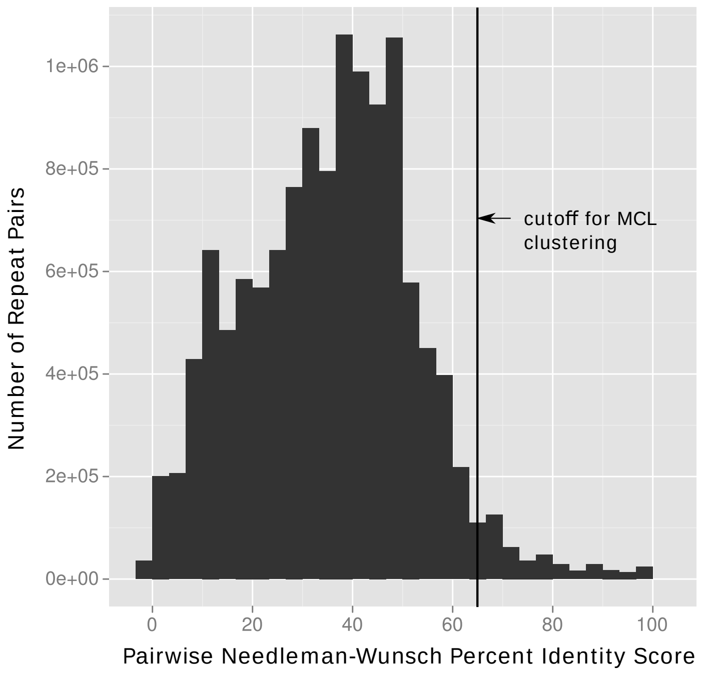

Figure S1: **Pairwise similarities for repeats.** We plotted the distribution of pairwise percent identities (x-axis) of Needleman-Wunsch [22] alignments for all repeats to determine a cutoff for the Markov clustering. Here we see that 65% is a reasonable cutoff in comparison to the background distribution. Repeats with a similarity below 65% are set to zero. Because of the short repeat length and conserved sequence motifs, it is necessary to choose such a high cutoff.

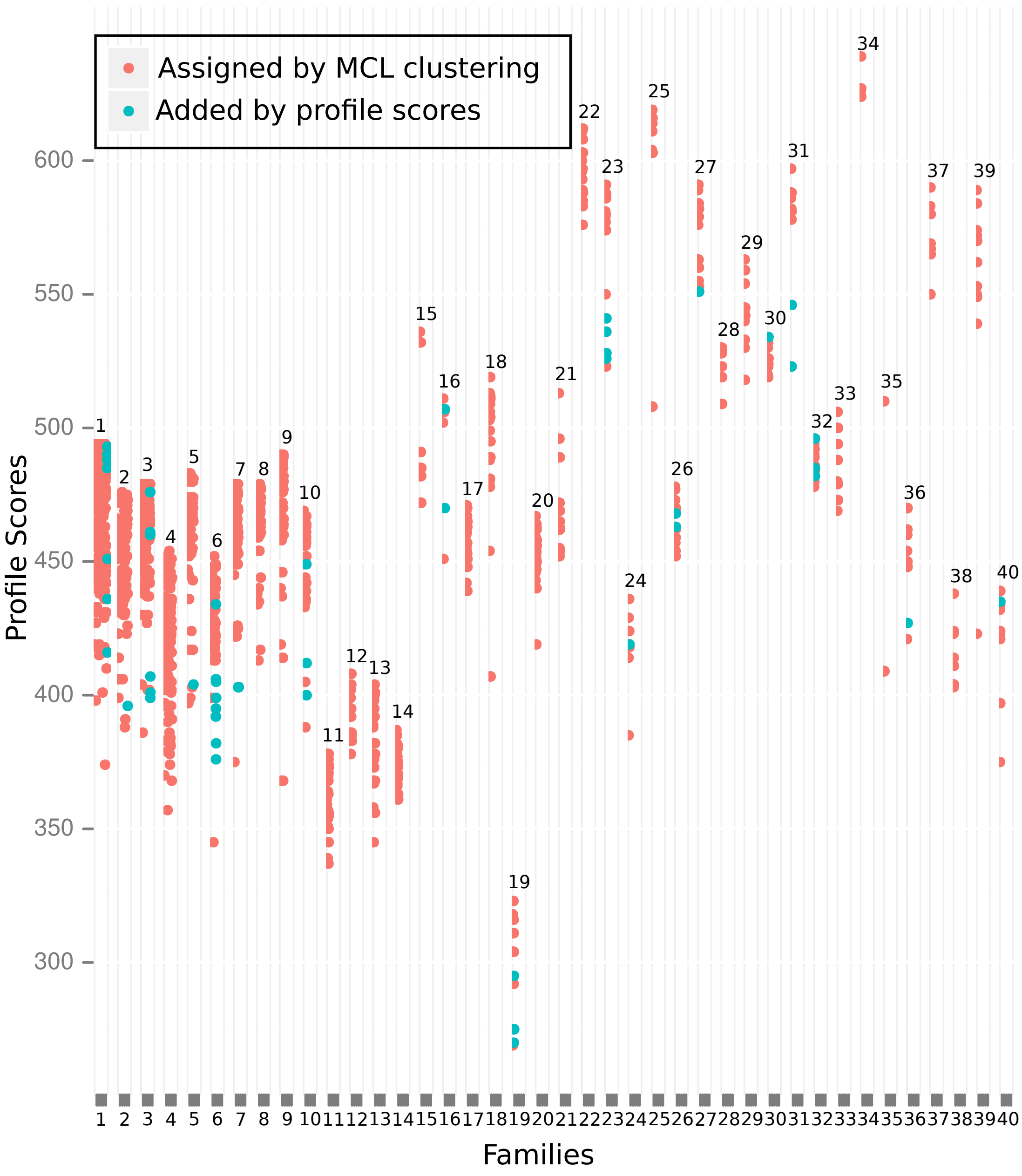

Figure S2: **Verifying repeat families with sequence profiles and re-assigning individual repeats.** All repeats were clustered into families using Markov clustering [23,24]. We verified these families using an independent method of sequence profiles, see Methods section "Clustering of repeat sequences into conserved sequence families". After the generation of one profile per family, we caculated the profile scores for each repeat in the REPEATS dataset. We plotted the profile scores (y-axis) for each repeat assigned to one of the families (x-axis) as red-coloured dots in Supplementary Figure S2. Subsequently, we used this range of profile scores to re-assign repeats to one of the existing families as stated in the main text of the manuscript. Profile scores for re-assigned dots are in blue (73 repeats). These profile scores are also used to assign new input repeat sequences from the webserver to one of our existing families.

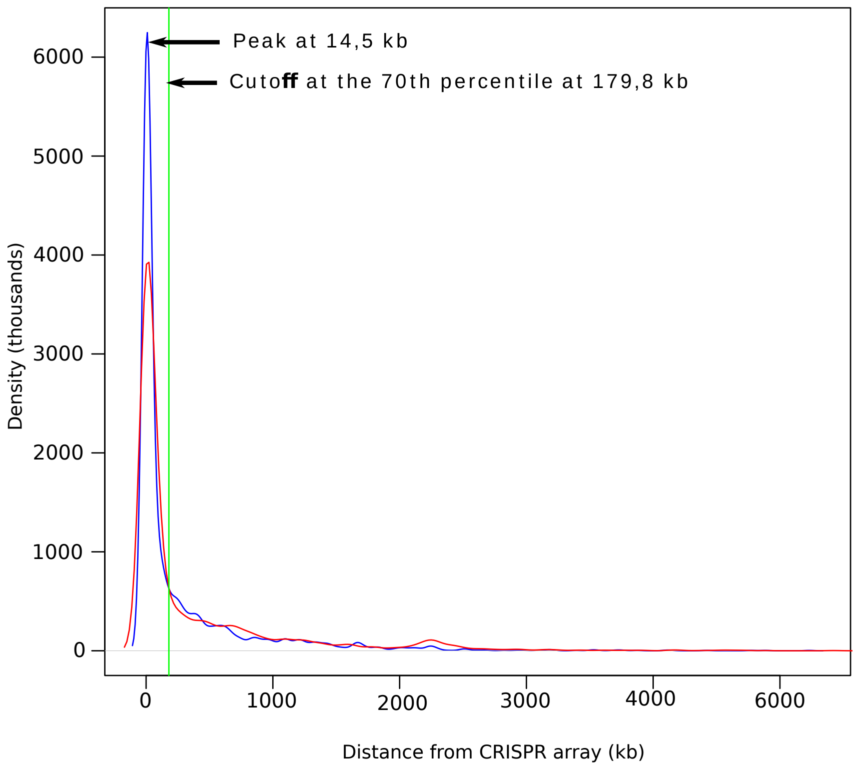

Figure S3: **Distance of *cas* genes in the annotation of subtypes from Makarova *et al.* 2011.** Distance of signature subtypes is in blue and the distance of signature types is in red; the cutoff is indicated with the green line. The plot shows the distribution of the closest signature genes to the CRISPR array. A signature gene is one that is unique to either the subtype or the type, respectively.

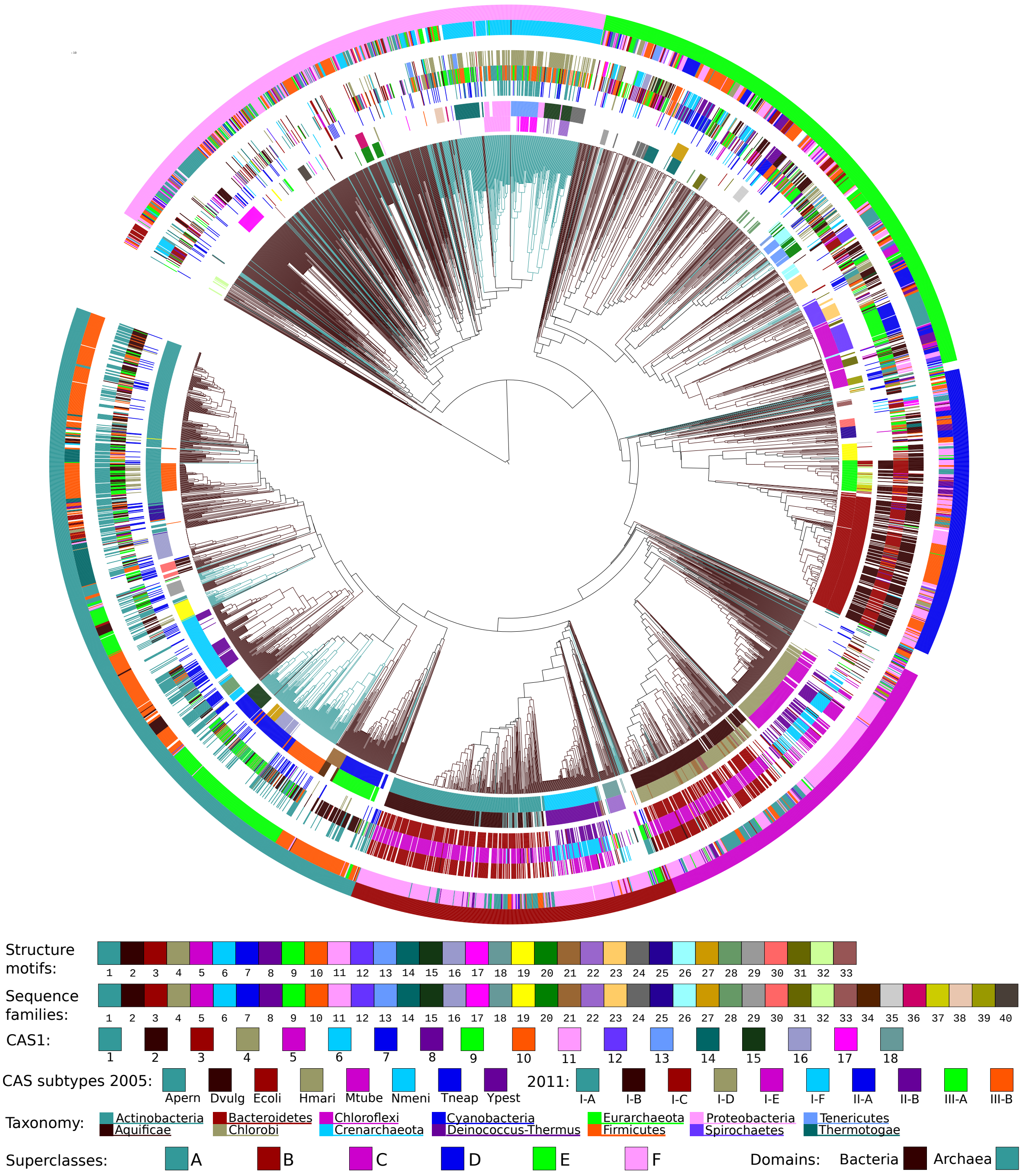

Figure S4: **CRISPR of repeat conservation including all annotations.** CRISPR repeats cluster into 33 structure motifs and 40 sequence families. Here we show the cluster tree with all annotation rings—the "alltogether" option in the webserver—colour coding starts from inside to outside, see the legend. The branches of the tree are labelled according to the origin of the repeat: blue-green for archaea and dark brown for bacteria. **Ring 1** (inner-most) 33 structure motifs, **ring 2** 40 sequence families, **ring 3** Haft 2005 subtype annotation, **ring 4** Makarova 2011 subtype annotation, **ring 5** 18 cas1 clusters, **ring 6** taxonomic phyla annotation and **ring 7** (outer-most) the six superclasses for general orientation.

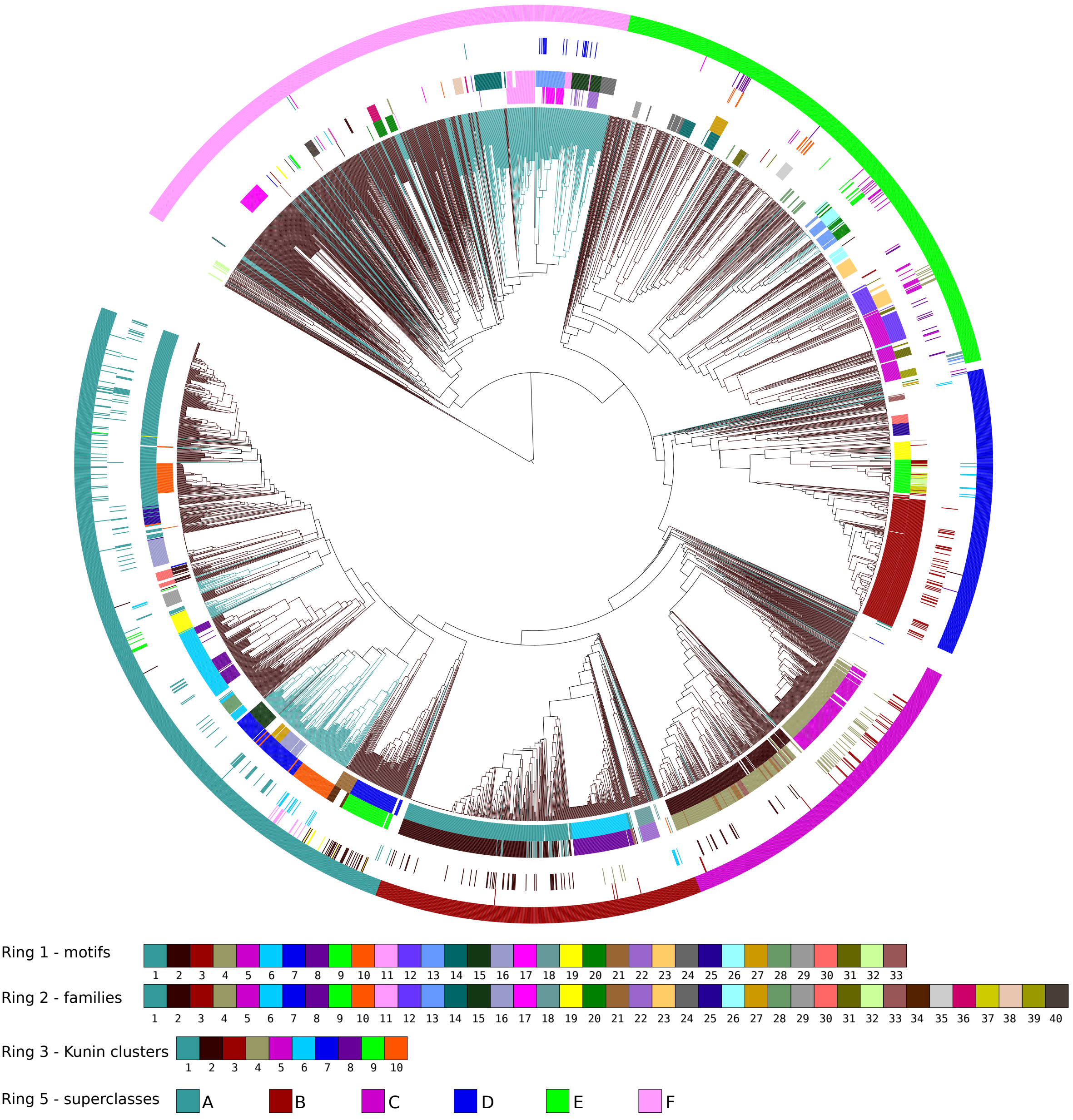

Figure S5: **Comparison of our clustering with previous domain-wide repeat clusters or families on our CRISPRmap tree.** The branches of the tree are labelled according to the origin of the repeat: blue-green for archaea and dark brown for bacteria. **Ring 1** (inner-most) shows our structure motifs, **ring 2** shows our sequence families. After the white ring, we show ten of the twelve clusters from Kunin *et al.* [2,25] in **Ring3**; clusters 11 and 12 contain fewer than ten repeats and to be consistent with our cluster minimum size, we have removed them here. **Ring 4** contains those sequences of the Rfam [26] database that are also contained in REPEATS (since we have all sequenced genomes to-date) and only families (16 out of 65) with at least ten sequences. We do not mark the family names here, but just want to show the relative locations of sequences in the CRISPRmap tree. **Ring 5** (outer-most) shows the six superclasses for general orientation. In summary, we clearly see that our data is significantly more comprehensive than previous work.

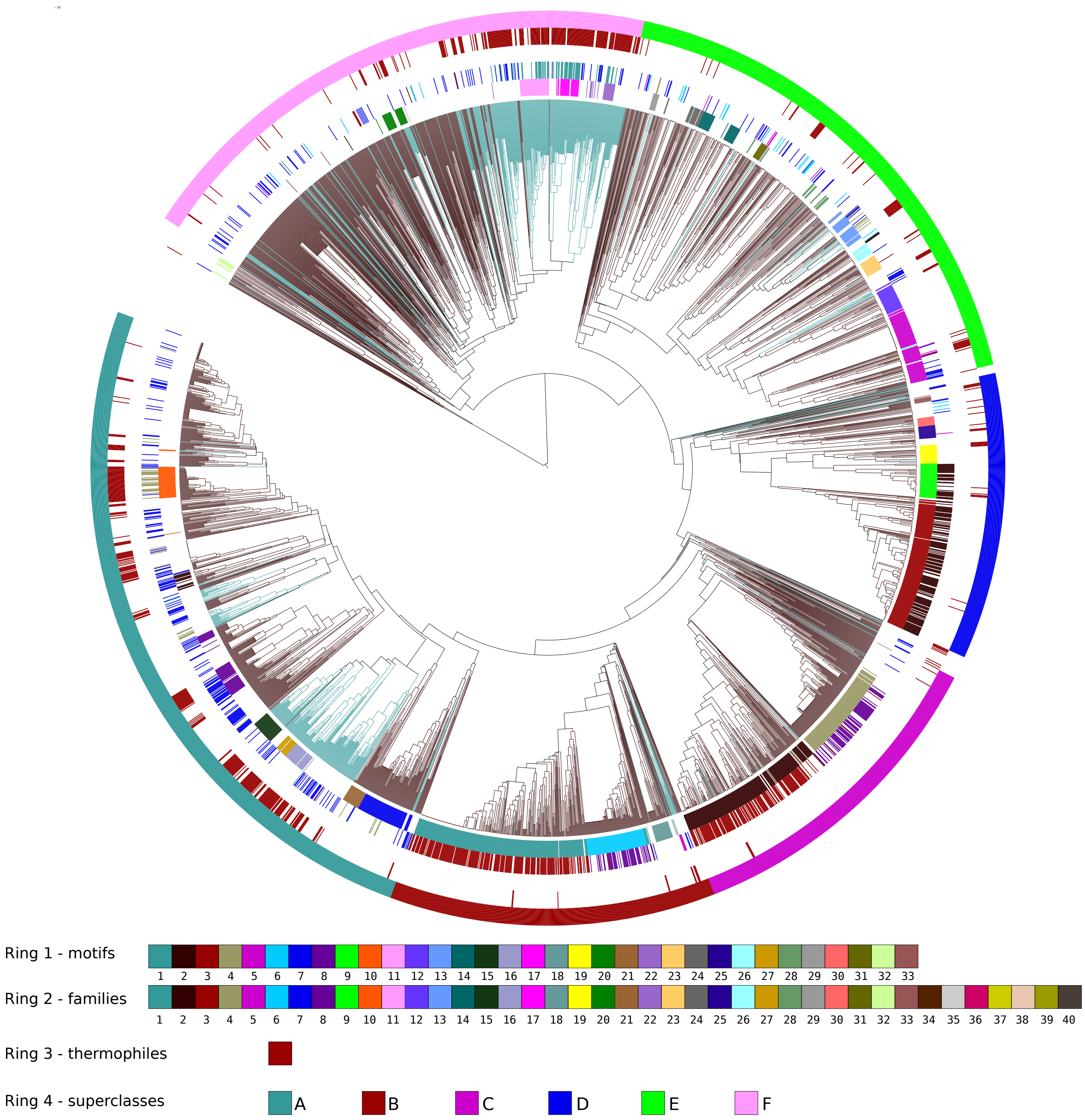

Figure S6: **CRISPRs found in thermophilic organisms. Ring 3** shows the number of CRISPRs that were found in thermophilic organisms (taken from ExtremeDB, `http://extrem.igib.res.in`, March 2013). At leat 17% of our CRISPRs stem from thermophiles. Of these CRISPRs, 81% are in superclasses A and F, which are associated with diverse types I-A, I-B, I-D, III-A and III-B. In contrast, only 7% of the bacterial CRISPRs in superclasses B, C, and D—with strong Cas subtype associations—stem from thermophiles. The same is true for bacteria only: 60% of the CRISPRs from bacterial thermophiles are in superclass A.

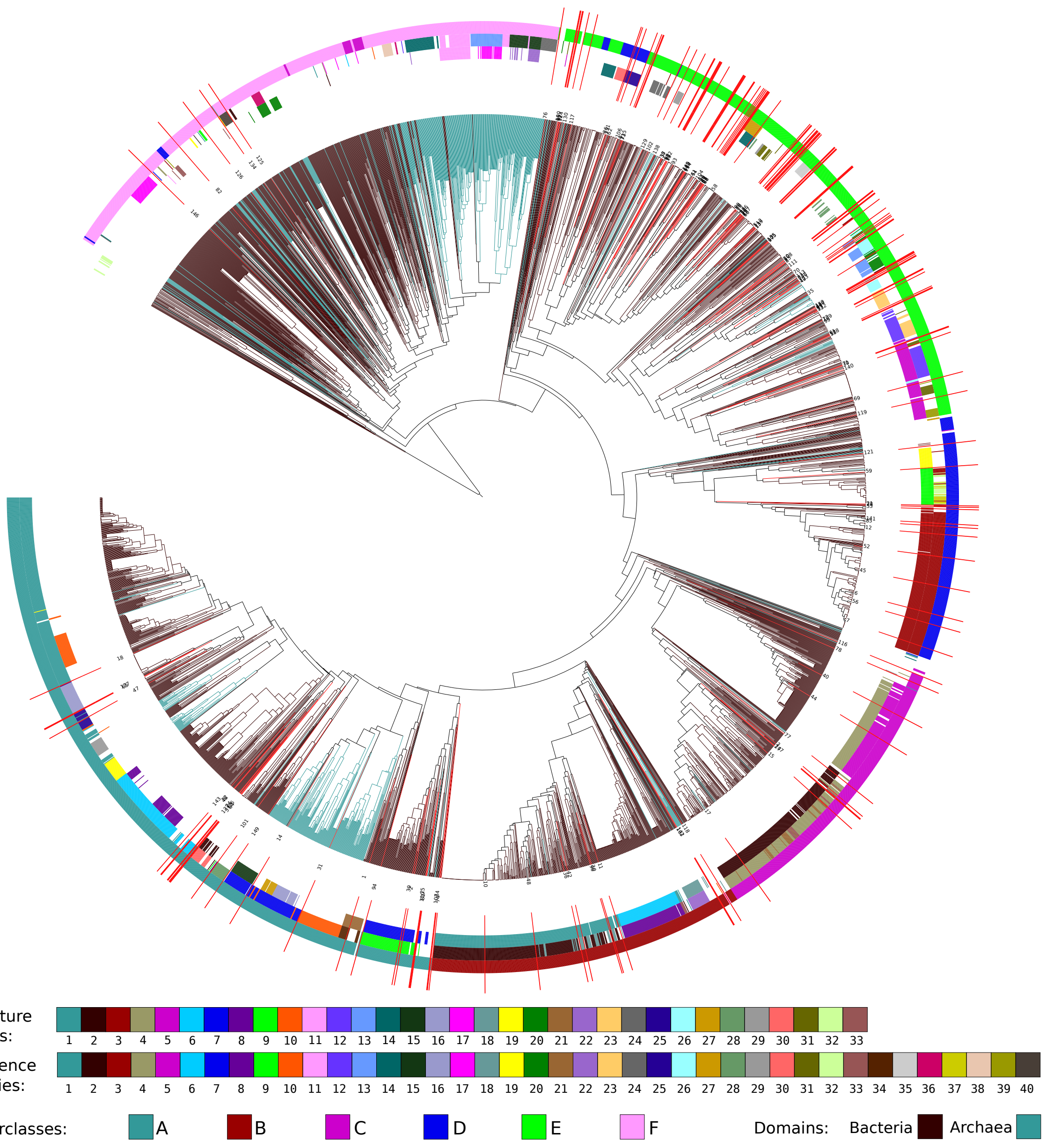

Figure S7: **CRISPRmap tree—a use-case study.** This is the CRISPRmap cluster tree after re-clustering 150 repeats from a human metagenomic studies [27] together with our REPEATS data. The new 150 repeats are maked with red lines. Interestingly, many repeats have been assigned to superclass E and cluster together to potentially form new classes of motifs or families.

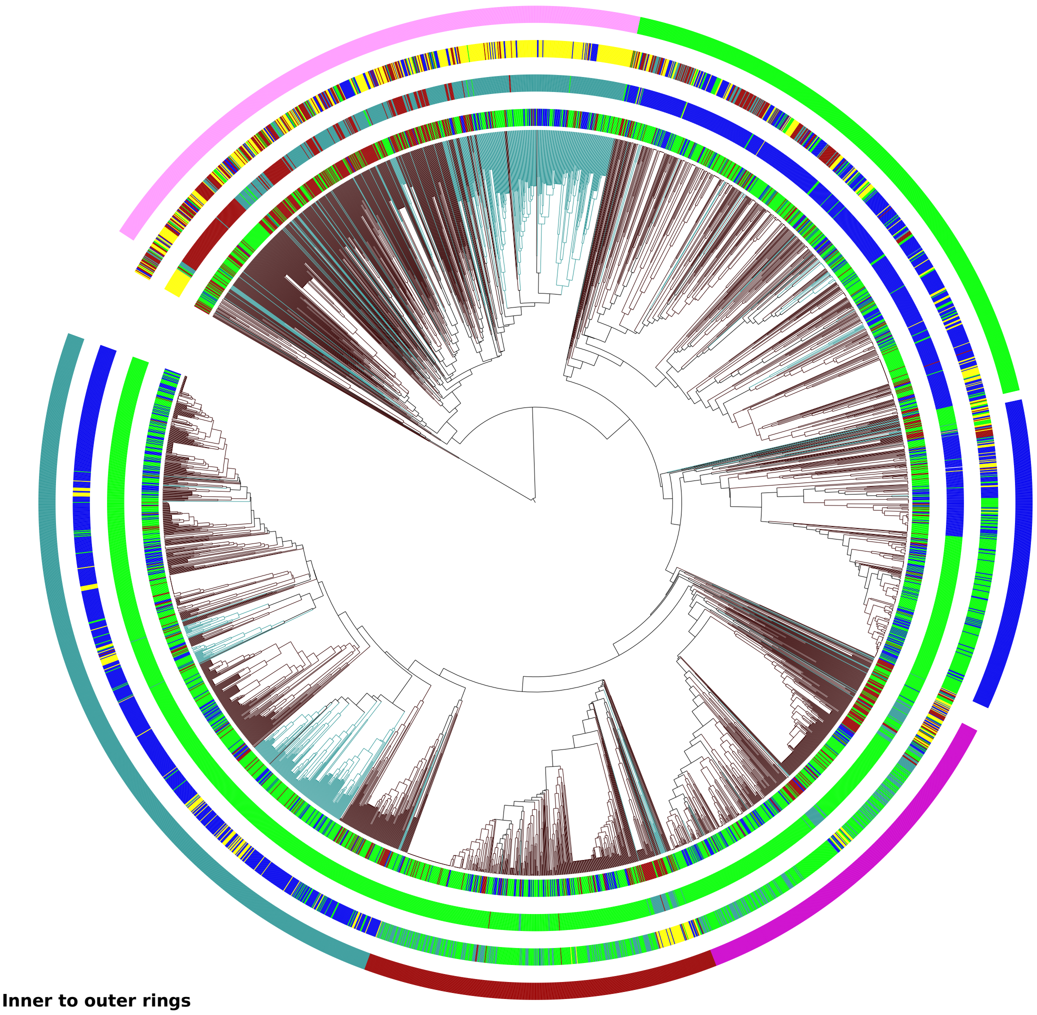

Figure S8: **Analysis of array, repeat and average spacer sizes.** First, we see the very small arrays containing less than 5 repeat instances (red-brown) are mostly located in the more divergent parts of the CRISPRmap tree; most are within the bacterial part of superclass F. Many of these arrays may not be functional CRISPR-Cas systems, but other repetitive elements instead. Second, superclass F contains both some unusually short and unusually long repeats, which also may not represent functional CRISPRs. In addition repeats in superclass F and half of D are longer than those in superclasses A to the first half of D. Third, repeats in superclasses A and F are longer than ones in B-D; this means the Cas subytpes I-C, I-E, and I-F associate with shorter spacers than the others. Spacers in Crenarchaeota are unusually long with most longer than 38 nt. Interestingly, shorter repeats seem to pair with longer spacers. Cutoffs were choses according to the distribution of each array characteristic.

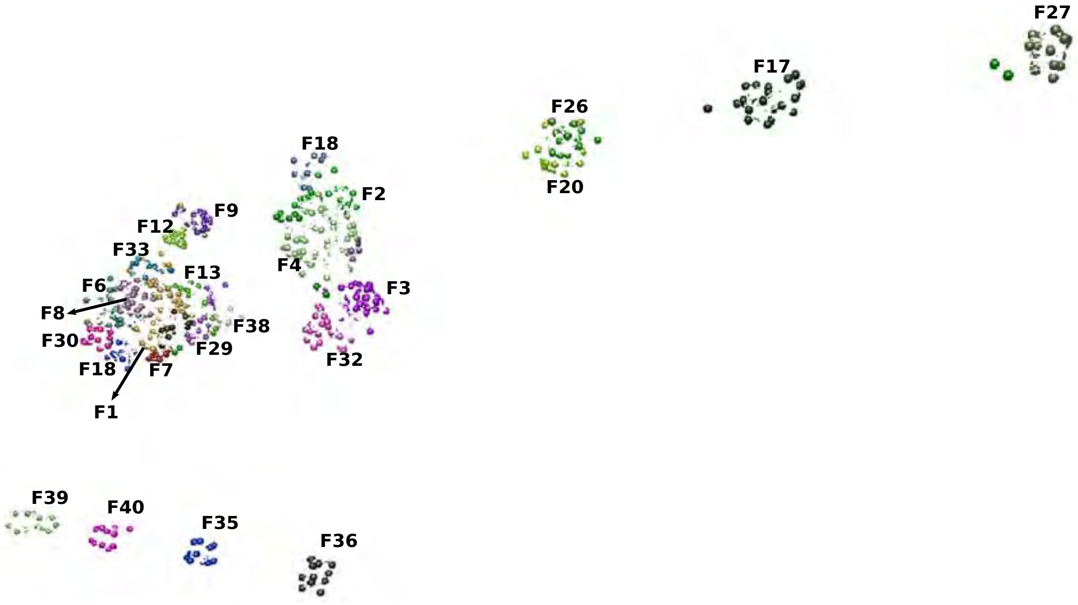

Figure S9: **Sequence families separated on a two-dimensional plane.** The 40 sequence famlies are mapped onto a two-dimensional plane by BioLayout [28] according to their percent identity scores. We have marked only those families that are clearly visible. The families are divided into two main groups with some that are more separated from the rest.

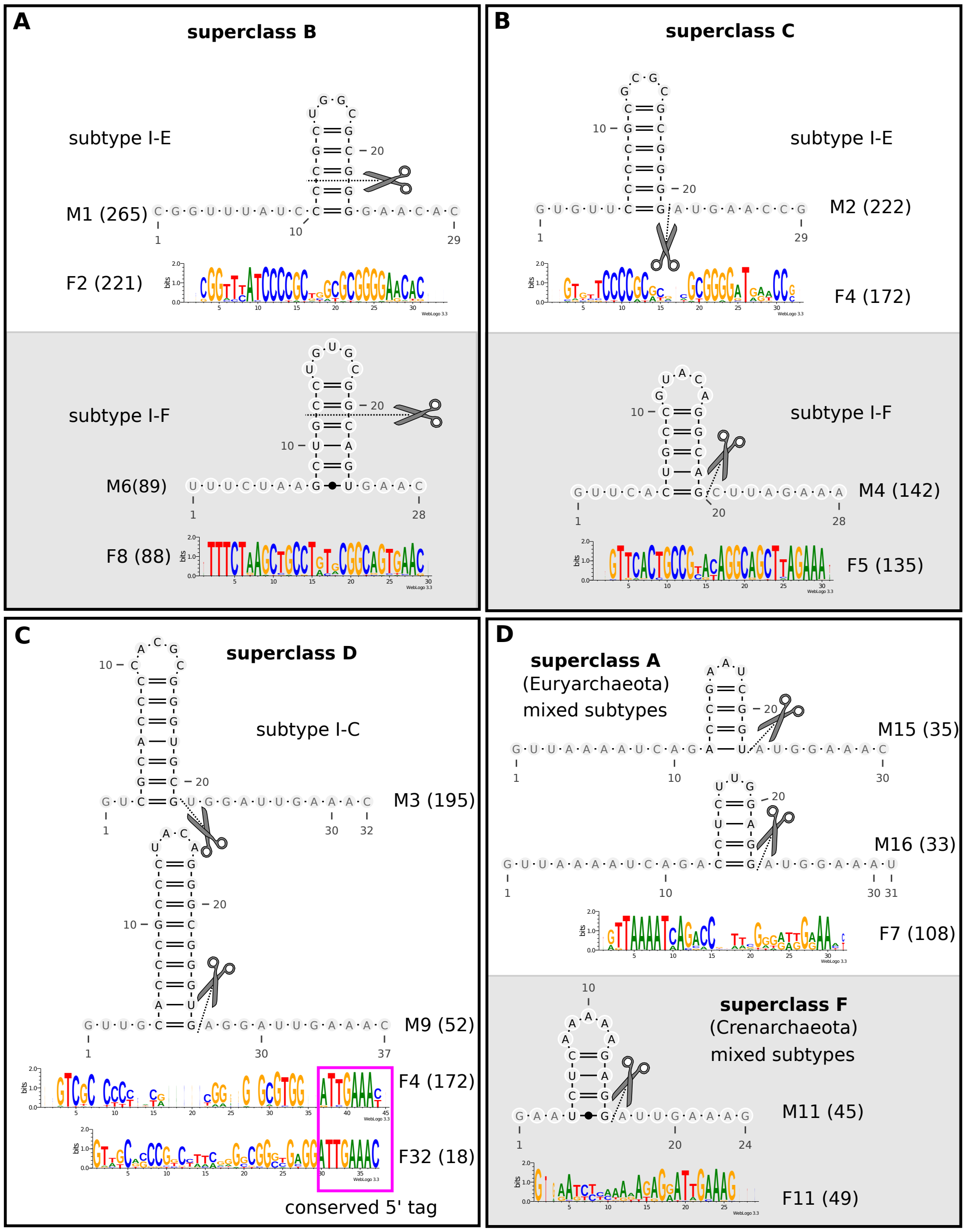

Figure S10: **Conserved structured CRISPRs fit well to published cleavage sites and display various patterns of sequence conservation.** The sequence family logos correspond to the depicted structure motifs. Potential cleavage sites are indicated as observed in the literature [**?**, 7–13, 15–18, 20]. **a.-b.** Superclasses B and C contain stable structure motifs of the subtypes I-E and I-F. The difference is that the structures in superclass B are closer to the 3' end of the repeat and that the potential cleavage site is in the double-stranded region of the stem instead of the 3' side of its base. **c.** Superclass D contains members of the I-C subtype with relatively long hairpin motifs. Note that the potential cleavage site leads to an 11 nt instead of an 8 nt tag in the mature crRNA and we also see the well-conserved 3' end of the repeat (*ATTGAAAC*); this 3' sequence is found in many CRISPRs, also in archaea. **d.** Examples of structure motifs found in archaeal repeats in superclasses A and F. These are smaller and less stable than the bacterial motifs.

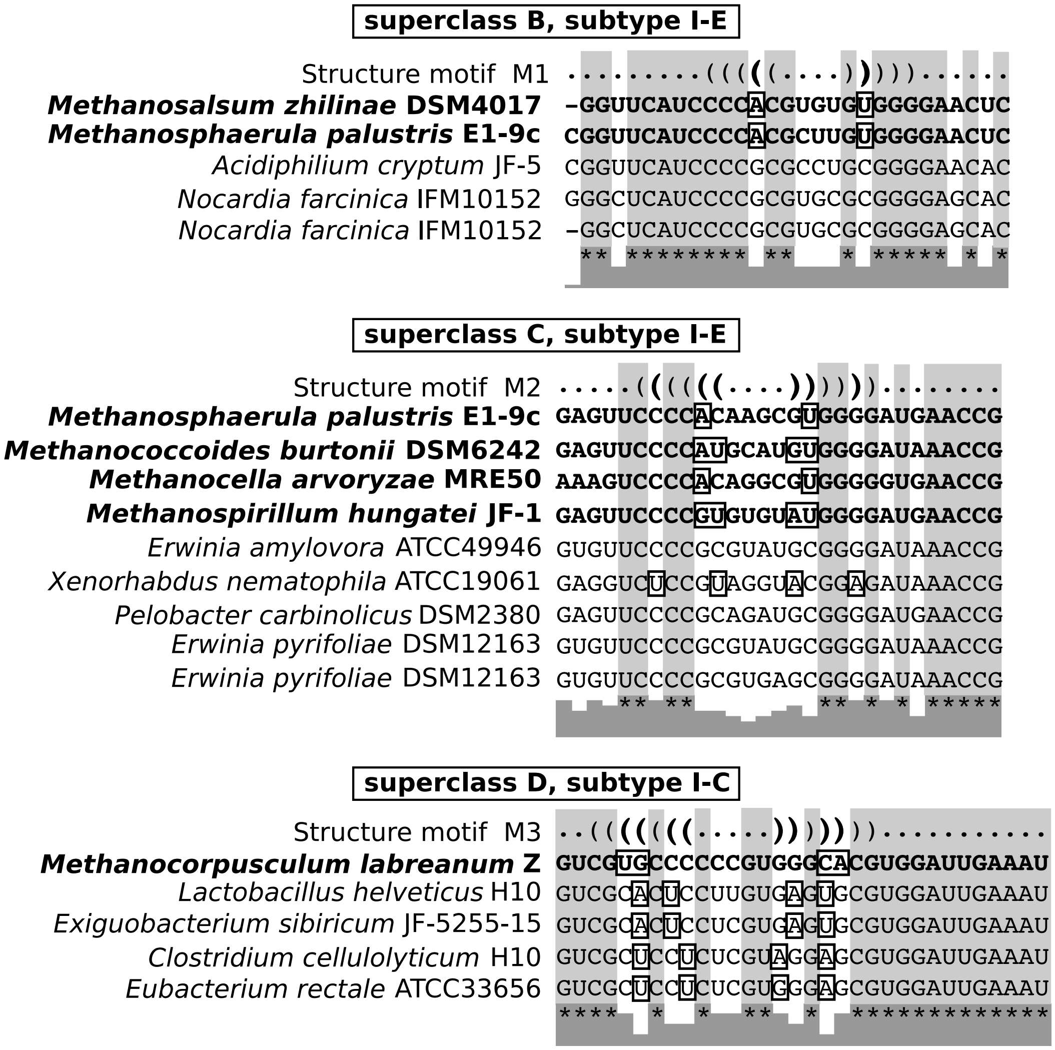

Figure S11: **Selected alignments showing evidence of horizontal transfer of structured CRISPRs from bacterial to archaeal genomes.** Archaeal CRISPRs are indicated in **bold** typeface. The secondary structure from the respective motif is written above in dot-bracket format: brackets and dots corresponds to base pairs and unpaired nucleotides, respectively. The highlighted brackets and squares show that the secondary RNA structure has been conserved by compensatory base pair mutations. These compensatory base pair mutations give excellent evidence for the conservation and importance of the respective structure motifs.

References

1. Haft DH, Selengut J, Mongodin EF, Nelson KE: **A guild of 45 CRISPR-associated (Cas) protein families and multiple CRISPR/Cas subtypes exist in prokaryotic genomes**. *PLoS Comput. Biol.* 2005, **1**(6):e60.
2. Kunin V, Sorek R, Hugenholtz P: **Evolutionary conservation of sequence and secondary structures in CRISPR repeats**. *Genome Biol.* 2007, **8**(4):R61.
3. Hofacker IL, Stadler PF: **Memory efficient folding algorithms for circular RNA secondary structures**. *Bioinformatics* 2006, **22**(10):1172–6.
4. Will S, Reiche K, Hofacker IL, Stadler PF, Backofen R: **Inferring Non-Coding RNA Families and Classes by Means of Genome-Scale Structure-Based Clustering**. *PLoS Comput. Biol.* 2007, **3**(4):e65.
5. Thompson JD, Higgins DG, Gibson TJ: **CLUSTAL W: improving the sensitivity of progressive multiple sequence alignment through sequence weighting, position-specific gap penalties and weight matrix choice**. *Nucleic Acids Res.* 1994, **22**(22):4673–80.
6. Makarova KS, Haft DH, Barrangou R, Brouns SJJ, Charpentier E, Horvath P, Moineau S, Mojica FJM, Wolf YI, Yakunin AF, van der Oost J, Koonin EV: **Evolution and classification of the CRISPR-Cas systems**. *Nat. Rev. Microbiol.* 2011, **9**(6):467–77.
7. Richter H, Zoephel J, Schermuly J, Maticzka D, Backofen R, Randau L: **Characterization of CRISPR RNA processing in Clostridium thermocellum and Methanococcus maripaludis**. *Nucleic Acids Res.* 2012.
8. Wang R, Preamplume G, Terns MP, Terns RM, Li H: **Interaction of the Cas6 riboendonuclease with CRISPR RNAs: recognition and cleavage**. *Structure* 2011, **19**(2):257–64.
9. Brouns SJJ, Jore MM, Lundgren M, Westra ER, Slijkhuis RJH, Snijders APL, Dickman MJ, Makarova KS, Koonin EV, van der Oost J: **Small CRISPR RNAs guide antiviral defense in prokaryotes**. *Science* 2008, **321**(5891):960–4.
10. Gesner EM, Schellenberg MJ, Garside EL, George MM, Macmillan AM: **Recognition and maturation of effector RNAs in a CRISPR interference pathway**. *Nat. Struct. Mol. Biol.* 2011, **18**(6):688–92.
11. Sashital DG, Jinek M, Doudna JA: **An RNA-induced conformational change required for CRISPR RNA cleavage by the endoribonuclease Cse3**. *Nat. Struct. Mol. Biol.* 2011, **18**(6):680–7.
12. Haurwitz RE, Jinek M, Wiedenheft B, Zhou K, Doudna JA: **Sequence- and structure-specific RNA processing by a CRISPR endonuclease**. *Science* 2010, **329**(5997):1355–8.
13. Haurwitz RE, Sternberg SH, Doudna JA: **Csy4 relies on an unusual catalytic dyad to position and cleave CRISPR RNA**. *EMBO J.* 2012, **31**(12):2824–32.
14. Sternberg SH, Haurwitz RE, Doudna JA: **Mechanism of substrate selection by a highly specific CRISPR endoribonuclease**. *RNA* 2012, **18**(4):661–72.
15. Nam KH, Haitjema C, Liu X, Ding F, Wang H, DeLisa MP, Ke A: **Cas5d protein processes pre-crRNA and assembles into a cascade-like interference complex in subtype I-C/Dvulg CRISPR-Cas system**. *Structure* 2012, **20**(9):1574–84.
16. Garside EL, Schellenberg MJ, Gesner EM, Bonanno JB, Sauder JM, Burley SK, Almo SC, Mehta G, MacMillan AM: **Cas5d processes pre-crRNA and is a member of a larger family of CRISPR RNA endonucleases**. *RNA* 2012, **18**(11):2020–8.
17. Randau L: **RNA processing in the minimal organism Nanoarchaeum equitans**. *Genome Biol.* 2012, **13**(7):R63.
18. Scholz I, Lange SJ, Hein S, Hess WR, Backofen R: **CRISPR-Cas Systems in the Cyanobacterium Synechocystis sp. PCC6803 Exhibit Distinct Processing Pathways Involving at Least Two Cas6 and a Cmr2 Protein**. *PLoS One* 2013, **8**(2):e56470.
19. Nickel L, Weidenbach K, Jager D, Backofen R, Lange SJ, Heidrich N, Schmitz RA: **Two CRISPR-Cas systems in Methanosarcina mazei strain Go1 display common processing features despite belonging to different types I and III**. *RNA Biol* 2013, **10**(5).

20. Hatoum-Aslan A, Maniv I, Marraffini LA: **Mature clustered, regularly interspaced, short palindromic repeats RNA (crRNA) length is measured by a ruler mechanism anchored at the precursor processing site**. *Proc. Natl. Acad. Sci. USA* 2011, **108**(52):21218–22.

21. Deltcheva E, Chylinski K, Sharma CM, Gonzales K, Chao Y, Pirzada ZA, Eckert MR, Vogel J, Charpentier E: **CRISPR RNA maturation by trans-encoded small RNA and host factor RNase III**. *Nature* 2011, **471**(7340):602–7.

22. Needleman SB, Wunsch CD: **A general method applicable to the search for similarities in the amino acid sequence of two proteins**. *J. Mol. Biol.* 1970, **48**(3):443–53.

23. van Dongen S: **Graph Clustering by Flow Simulation**. *PhD thesis*, University of Utrecht 2000.

24. Enright AJ, Van Dongen S, Ouzounis CA: **An efficient algorithm for large-scale detection of protein families**. *Nucleic Acids Res.* 2002, **30**(7):1575–84.

25. Shah SA, Garrett RA: **CRISPR/Cas and Cmr modules, mobility and evolution of adaptive immune systems**. *Res. Microbiol.* 2011, **162**:27–38.

26. Gardner PP, Daub J, Tate J, Moore BL, Osuch IH, Griffiths-Jones S, Finn RD, Nawrocki EP, Kolbe DL, Eddy SR, Bateman A: **Rfam: Wikipedia, clans and the "decimal" release**. *Nucleic Acids Res.* 2011, **39**(Database issue):D141–5.

27. Rho M, Wu YW, Tang H, Doak TG, Ye Y: **Diverse CRISPRs evolving in human microbiomes**. *PLoS Genet.* 2012, **8**(6):e1002441.

28. Theocharidis A, van Dongen S, Enright AJ, Freeman TC: **Network visualization and analysis of gene expression data using BioLayout Express(3D)**. *Nat. Protoc.* 2009, **4**(10):1535–50.
